# Genetic diversity and population structure of *Lactobacillus delbrueckii* subspecies *bulgaricus* isolated from naturally fermented dairy foods

**DOI:** 10.1038/srep22704

**Published:** 2016-03-04

**Authors:** Yuqin Song, Zhihong Sun, Chenyi Guo, Yarong Wu, Wenjun Liu, Jie Yu, Bilige Menghe, Ruifu Yang, Heping Zhang

**Affiliations:** 1 Key Laboratory of Dairy Biotechnology and Engineering, Education Ministry of China, Inner Mongolia Agricultural University, Hohhot, Inner Mongolia 010018, China; 2 State Key Laboratory of Pathogen and Biosecurity, Beijing Institute of Microbiology and Epidemiology, Beijing 100071, China

## Abstract

*Lactobacillus delbrueckii* subsp. *bulgaricus* is one of the most widely used starter culture strains in industrial fermented dairy manufacture. It is also common in naturally fermented dairy foods made using traditional methods. The subsp. *bulgaricus* strains found in naturally fermented foods may be useful for improving current industrial starter cultures; however, little is known regarding its genetic diversity and population structure. Here, a collection of 298 *L*. *delbrueckii* strains from naturally fermented products in Mongolia, Russia, and West China was analyzed by multi-locus sequence typing based on eight conserved genes. The 251 confirmed subsp. *bulgaricus* strains produced 106 unique sequence types, the majority of which were assigned to five clonal complexes (CCs). The geographical distribution of CCs was uneven, with CC1 dominated by Mongolian and Russian isolates, and CC2–CC5 isolates exclusively from Xinjiang, China. Population structure analysis suggested six lineages, L1–L6, with various homologous recombination rates. Although L2–L5 were mainly restricted within specific regions, strains belonging to L1 and L6 were observed in diverse regions, suggesting historical transmission events. These results greatly enhance our knowledge of the population diversity of subsp. *bulgaricus* strains, and suggest that strains from CC1 and L4 may be useful as starter strains in industrial fermentation.

*Lactobacillus delbrueckii* subsp. *bulgaricus*, a Gram-positive, rod-shaped, non-motile and non-spore-forming lactic acid bacterium (LAB), is one of the most common starter bacterial species in industrial fermentation of dairy products. Globally, almost all types of yogurt and a considerable proportion of other types of dairy products contain this bacterium. It is also an important component in the traditional production of naturally fermented dairy products, which generally use bacteria obtained from the local environment^1^. Previous reports have shown that many different LAB coexist during fermentation of traditional dairy products, contributing to their special flavor and texture^1^,^2^,^3^,^4^. Although the dominant species of LAB varies in different dairy products (e.g. *Lactobacillus helveticus*, *Lactobacillus kefiranofaciens*, and *L. delbrueckii* were the predominant LAB species in airag, khoormog, and tarag, respectively^4^), *L. delbrueckii* subsp. *bulgaricus* was identified as the predominant bacterial strain in certain types of naturally fermented products in China and Mongolia^1^,^2^.

Naturally fermented dairy products have a long history in China, Mongolia, and Russia. A book titled *A New Account of Tales of the World (Shi Shuo Xin Yu)* was published in the Eastern Jin Dynasty in China (AD 317–420), documenting the production of a fermented dairy product named “lao”. Nomadic people across large rural areas of these countries still use traditional methods to produce fermented dairy products. These products have tremendous potential as sources of novel subsp. *bulgaricus* strains for improving industrial starter cultures. Improving our understanding of the diversity and phylogenetic relationships of subsp. *bulgaricus* isolates from naturally fermented products would provide insights into the evolutionary patterns of this subspecies, and would support more effective selection of the next generation of starter strains for industrial applications.

Different molecular methods have been reported for the identification and typing of *L. delbrueckii*, including randomly amplified polymorphic DNA analysis^5^, sodium dodecyl sulfate polyacrylamide gel electrophoresis^6^, restriction fragment length polymorphism analysis^7^, amplified fragment length polymorphism analysis^8^, and multi-locus sequence typing (MLST)^9^,^10^,^11^. While all of these methods can distinguish subsp. *bulgaricus* from other subspecies, including subsp. *delbrueckii*, subsp. *lactis*^12^, subsp. *indicus*^8^, subsp*. sunkii*^10^, and subsp. *jakobsenii*^11^, MLST is generally the preferred method because of its convenience and higher resolution, and the ease of data interchange between global laboratories^13^. However, only three MLST studies associated with subsp. *bulgaricus* have been published, and all employ a limited number of strains. Therefore, our knowledge of the genetic diversity and population structure of subsp. *bulgaricus* strains still remains limited.

In this study, we collected 298 *L. delbrueckii* strains (287 strains identified by 16S rRNA sequencing obtained from naturally fermented dairy foods in traditional pasturing areas, and 11 strains retrieved from the NCBI GenBank database), with subsp. *bulgaricus* accounting for 84% of isolates. We calculated the diversity and predicted the population structure of the strains based on a newly developed MLST scheme. From these results, we inferred the geographical distribution and recombination characteristics of different lineages in an attempt to provide information that will be useful in starter strain selection in the fermentation industry.

## Results

### MLST assay for identifying *L. delbrueckii* subsp. *bulgaricus*

A total of 298 strains were analyzed using the amplified fragments of eight housekeeping genes (*clpX, dnaA, groEL, murE, pheS, pyrG, recA,* and *rpoB*) (Supplementary Table S1). All gene fragments were successfully amplified from all strains tested. Our eight-gene assay shared a single locus, *pheS*, with a previously reported three-gene MLST assay^9^, and shared three loci (*groEL/hsp60, pyrG, recA*) with a seven-locus scheme^10^.

The *d*_*N*_*/d*_*S*_ ratio is a common indicator of selection pressure, with *d*_*N*_*/d*_*S*_ > 1 indicating positive selection and *d*_*N*_*/d*_*S*_ < 1 indicating purifying selection for the gene sequence tested^14^. For each of the eight loci, the calculated *d*_*N*_*/d*_*S*_ ratios were all less than one, especially for *recA*, where *d*_*N*_*/d*_*S*_ = 0 (no nonsynonymous single nucleotide polymorphisms (SNPs) were found) (Table 1). This indicated that all loci were under strong purifying selection, which is optimal for MLST loci^15^.

### Definition of *L. delbrueckii* subsp. *bulgaricus*

All isolates included in this study were previously identified as *L. delbrueckii* by 16S RNA gene sequencing (data not shown). However, with poor distinguishing power, 16S RNA-based typing methods cannot accurately identify strains to the subspecies level. Therefore, we included the sequences of type strains of each subspecies (subsp. *bulgaricus*: ATCC 11842^T^, subsp. *delbrueckii*: JCM 1012^T^, subsp. *lactis*: DSM20072^T^, subsp. *indicus* : DSM15966^T^, subsp. *jakobsenii*: ZN7a-9^T^, subsp. *sunkii*: DSM24966^T^, Table S2)^8^,^11^,^16^,^17^ in the analysis as reference strains to more accurately identify isolates belonging to subsp. *bulgaricus*.

The maximum likelihood tree based on the concatenated sequences of the eight loci showed that the six type strains formed distinct lineages (Supplementary Fig. S1). Most of the tested strains (249 of 298) grouped together with the subsp. *bulgaricus* type strain (ATCC 11842^T^) to form a distinct branch. Forty-five strains were genetically distant from subsp. *bulgaricus*, and were more closely related to the other five type strains. Three of the strains (IMAU90010, IMAU90013, and IMAU94251) were located at an intermediate position between the subsp. *bulgaricus* lineage and the other lineages. A neighbor-joining tree was then generated for each locus separately, and again, 249 of the strains clustered with the subsp. *bulgaricus* type strain (Supplementary Fig. S2). Only six, four, and three loci supported the grouping of the three intermediate strains, IMAU90010, IMAU90013, and IMAU94251, respectively, with subsp. *bulgaricus*. The sequences of the remaining loci were more closely related to other subspecies, which may indicate inter-lineage homologous recombination within the species. As supported by the majority of the loci, we included the strain IMAU90010 as subsp. *bulgaricus* in further analyses.

Therefore, 251 strains were identified as subsp. *bulgaricus* in this study. Among the 246 subsp. *bulgaricus* isolates obtained from dairy products, 190 (77.2%) were from fermented cow milk, and 45 (18.3%) were from fermented yak milk (Supplementary Table S2). Most of these strains were collected from Asia (China: 107, Mongolia: 117, Middle-South Russia: 19, Japan: 1), with the remaining strains originating from Denmark (NBIMCC1381), France (ATCC BAA-365), North America (CNCM1519, CNCM1632), and Bulgaria (NBIMCC1273, NBIMCC285, and ATCC11842^T^) (Supplementary Table S2).

### Genetic diversity of subsp. *bulgaricus*

We aligned the sequences for each strain at each MLST locus and confirmed that neither indels nor premature stop codons were present in any of the sequences. The DNA fragments ranged from 470 bp (*groEL*) to 603 bp (*rpoB*), and the concatenated sequences produced a 4,261-bp fragment. Amongst the 298 *L. delbrueckii* strains, 20 (*dnaA*) to 27 (*groEL* and *pyrG*) alleles per locus were identified, corresponding to 119 STs. For the 251 subsp. *bulgaricus* strains, the alleles numbers varied from 12 (*dnaA*) to 20 (*groEL*), and 106 STs were assigned (Table 1). There were 113 polymorphic sites amongst the subsp. *bulgaricus* sequences, accounting for only half of the variation (224 sites) observed for the species *L*. *delbrueckii*. The nucleotide diversity (π, the average number of nucleotide differences per site between two randomly selected isolates) was also calculated. Within subsp. *bulgaricus*, the π values ranged from 0.0038 (*dnaA*) to 0.0069 (*pyrG*), while at the species level, the range was from 0.0056 (*rpoB*) to 0.0109 (*clpX*). Taking the eight loci together, the π value was 0.0030 for the subsp. *bulgaricus* lineage, and 0.0042 for the whole species. Overall, although lower than for the whole species, subsp. *bulgaricus* still displayed a high level of nucleotide and allelic diversity.

### Population snapshot

**Figure 1:**
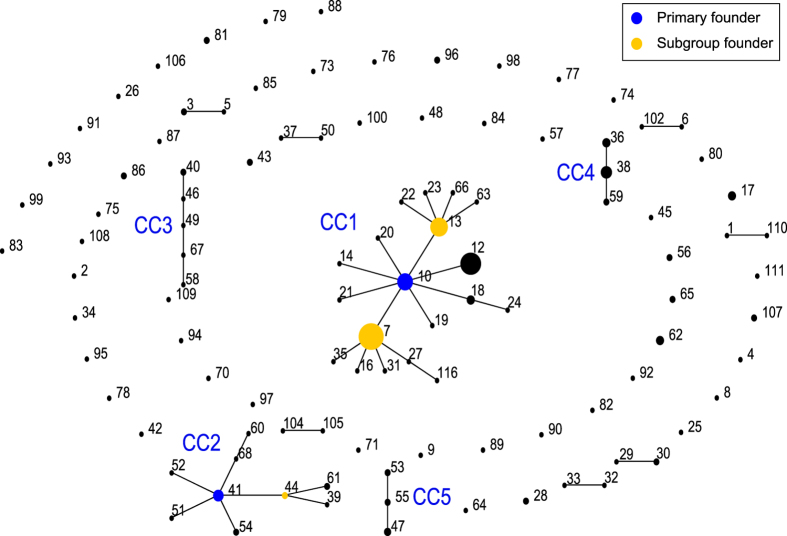
Population snapshot generated by eBURST based on the allelic profiles of 251 subsp. *bulgaricus* isolates. Each dot indicates an individual sequence type (ST), with the size of the dot representing the number of strains. The numbers associated with the dots represent the assigned STs. Solid lines link single locus variants, i.e. STs identical at seven of the eight loci, to constitute the clonal complex.

The 251 subsp. *bulgaricus* strains were assigned 106 different STs, with the allele profiles listed in Supplementary Table S2. According to eBURST analysis, the 251 strains could be assigned into five clonal complexes (CC1–CC5, 168 strains), seven doubletons (16 strains), and 53 singletons (67 strains) (Fig. 1). CC1, containing almost half of all strains (n = 123), was the largest CC, and consisted of 19 STs. ST10, with eight single-locus variants (SLVs), was defined as the group founder. ST7 (accounting for the largest number of strains among the 106 STs) and ST13 were predicted to be subgroup founders of CC1. CC2 consisted of 18 strains within nine STs. The group founder, ST41, had five SLVs. CC3 included six strains with five STs (Supplementary Table S2). CC4 and CC5 contained 14 and seven strains, respectively, each with three STs. About one-third of strains (33%, singletons and doubletons) appeared to be less related to each other and the rest of the population.

### The whole population contains six lineages

**Figure 2:**
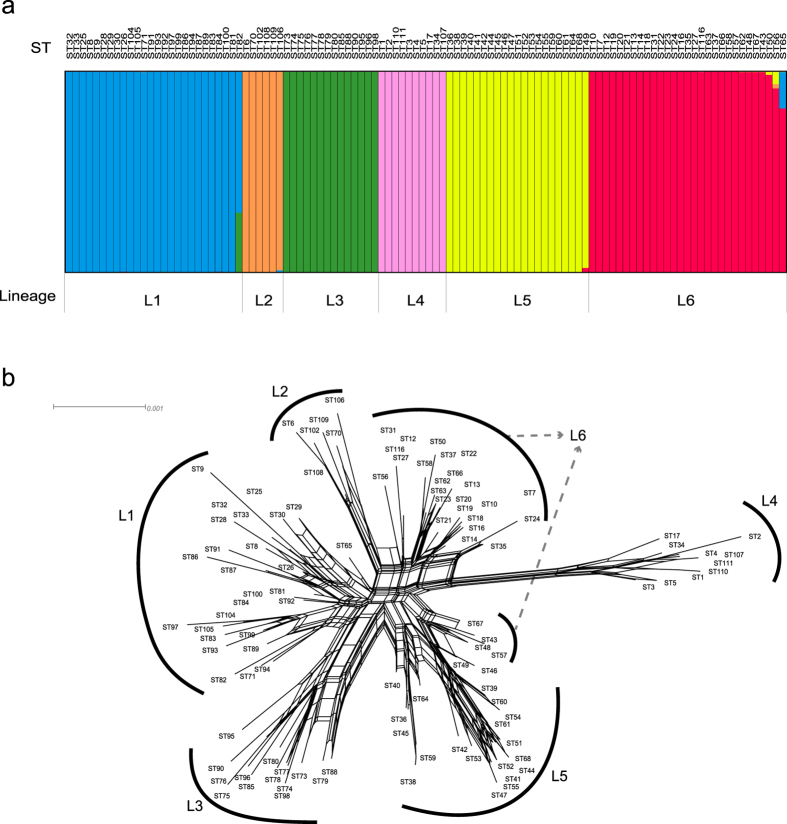
Population structure of subsp*. bulgaricus* based on concatenated sequences of the eight MLST loci. (**a**) Population structure inferred by Structure analysis. Each bar represents one of 106 sequence types (STs). Different colors indicate the predicted ancestor populations of subsp*. bulgaricus* as inferred by Structure analysis (assuming K = 6 and applying the linkage model). The proportion of the colors for each ST represents the ancestors’ contribution to the ST. Based on this, six lineages (L1–L6) are predicted, and given boundaries by the vertical gray lines. (**b**) Split network inferred by SplitsTree. Except for four STs, the six branches indicated by the black arcs are consistent with L1–L6 defined according to Structure analysis.

As shown in Supplementary Fig. S1, low bootstrap values on many branches of the maximum likelihood tree indicated uncertain phylogenetic relationships and the possible existence of homologous recombination. To identify sub-populations of subsp. *bulgaricus* that may have been affected by recombination, a linkage model generated by Structure analysis was applied to the concatenated sequences. The results of triple runs with K values from 3–20 were compared, and K = 6 was selected for use in further analyses. This suggested that the collected subsp. *bulgaricus* strains were descendants of six distinct ancestor populations. The rebuilding of population structure showed that the 106 STs fell into six distinct lineages (L1–L6), with little intersection of ancestral sources. The STs within each lineage tended to be highly homogenous (Fig. 2a).

To verify the population structure of the six lineages, we generated a split graph using SplitsTree based on the concatenated sequences of the 106 STs. Except for four STs belonging to L6 (ST43, ST48, ST57, ST67) that were closer to L5 in the split graph, the network-like structure with six branches was largely consistent with the six lineages defined by Structure analysis (Fig. 2b). Therefore, although a minority of intermediate types might exist, both Structure and SplitsTree analysis results supported the hypothesis that subsp. *bulgaricus* strains could be separated into six major lineages.

### The lineages show various recombination levels

The *phi*-test was used to give a statistical view of recombination^18^. The P value generated for all 106 STs was 0.0018 (Table 2), indicating significant evidence of recombination across the whole subspecies. However, P values for the six individual lineages varied, ranging from 0.0048–1 (Table 2). Only L3 and L6 showed significant intra-lineage recombination (both P values were <0.05). Correspondingly, higher ρ/θ ratios (the number of recombination events versus mutation events) were observed in L3 and L6 (39.969 and 17.587, Table 2), indicating recombination events occurred more frequently within these lineages.

The standardized index of association (*I*_*A*_^S^)^19^ was also calculated to measure the homologous recombination by estimating the linkage disequilibrium between the eight loci of each lineage (Table 2). In addition to lineages L3 and L6, the *I*_*A*_^S^ value for L4 also suggested a tendency towards frequent recombination between alleles in this lineage. The *I*_*A*_^S^ values for L1, L2, and L5 indicated significant linkage disequilibrium among the alleles, suggesting clonal relationships might dominate within these lineages.

### Population structure in relation to geographical distribution

**Figure 3:**
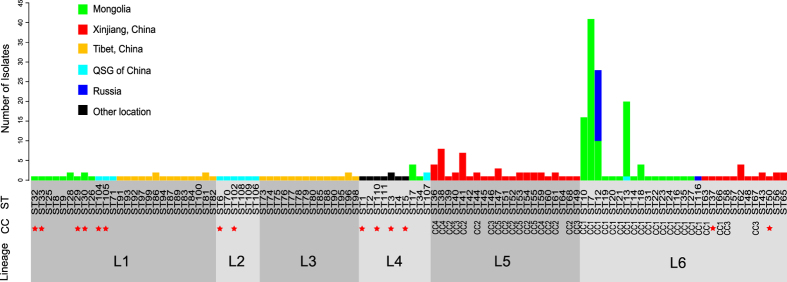
Geographical distribution of clonal complexes (CCs) and lineages of subsp*. bulgaricus*. Each bar represents one of the 106 sequence types (STs), of which the colors and height indicate the region of isolation and numbers of isolates, respectively. ST type and CC distribution (red stars for doubletons and blanks for singletons) are indicated below the bars. The gray shades indicate the six lineages defined by Structure analysis. “QSG of China” refers to Qinghai, Sichuan, and Gansu provinces of China, and “Other location” indicates Japan (ST1), Denmark and France (ST3), North America (ST4, ST5), and Bulgaria (ST2, ST110, ST111).

An unbalanced distribution of the six lineages of subsp. *bulgaricus* was observed among the sampling regions, with three lineages associated with a specific region, i.e. L2 in QSG of China, L3 in Tibet, and L5 in Xinjiang (Fig. 3). This geographical clustering may suggest possible adaptive advantages for these lineages in the local region. Comparatively, except for three STs isolated from QSG of China, approximately half of STs of L1 were isolated from Mongolia, while the other half of the lineage was from Tibet. A similar composition was also observed for L6, with half of the STs from Mongolia and the other half from Xinjiang, China. Therefore, transfer of the strains over long distances may have occurred during the evolutionary history of subsp. *bulgaricus*.

Interestingly, the distribution of the CCs also showed geographical dependency (Supplementary Fig. S3). Strains of the largest clonal complex, CC1, were all collected from Mongolia and Russia, except for two strains each from Xinjiang and QSG of China. The remaining four CCs only contained strains isolated from Xinjiang, China. Except for one strain from CC1, the strains from QSG of China formed four doubletons and seven singletons. Interestingly, all 28 STs (31 strains) from Tibet were singletons, although they were collected from a limited geographical area (Supplementary Table S2).

## Discussion

This study has greatly enhanced our knowledge of the genetic diversity of *L. delbrueckii* subsp. *bulgaricus*. We identified 106 STs and six lineages amongst the strains examined, while a previous study identified 15 STs and two lineages among 25 strains^9^. Our results also expand the known nucleotide diversity of the species *L. delbrueckii*, with increased π values compared with previous research observed for three loci (*pyrG*, 0.0090 vs. 0.0068; *groEL*/*hsp60*, 0.0081 vs. 0.0066; *recA*, 0.0079 vs. 0.0066)^10^. The MLST assay developed here may be useful in further determining the population structure of subsp. *bulgaricus*, but also in distinguishing subsp. *bulgaricus* from the other subspecies of *L. delbrueckii*. However, as a limited number of strains from non-*bulgaricus* subspecies were examined in this study, the typing efficiency of this assay for other subspecies of *L. delbrueckii* should be examined in further work.

Mongolia has one of the largest livestock husbandry industries in the world, and is famous for its dairy products. In this research, we collected subsp. *bulgaricus* strains from 13 of the 22 Mongolian provinces. The majority of the strains collected from Mongolia (101 of 117, 86%) belonged to CC1 (Fig. 3), suggesting that strains of this clonal complex had selective advantages in producing naturally fermented dairy foods in Mongolia. CC1 also had the richest diversity, with 19 STs and 123 strains, and was found across a much wider area, including South-west Russia (regions adjoining Mongolia), Xinjiang, and QSG of China, implying strong adaptability in different niches by these strains. Although any genetic mechanisms accounting for this selective advantage still need to be illuminated, probably by whole genome sequencing, strains of CC1 could potentially be used for improving current industrial starter cultures. Additionally, all strains from industrial dairy products or their descendants (ATCC BAA-365, 2038, CNCM I-1519, CNCM I-1632, NBIMCC1273, NBIMCC1381)^20^,^21^,^22^ were grouped in L4. Any of the other isolates in this lineage would likely display similar characters in fermentation of dairy products, and could therefore be another potential source of new starter culture strains. It is notable that many lactobacilli coexist in the natural fermented dairy foods, and both the species composition and their relative abundance contribute to the special flavor and texture. Therefore, although CC1 and L4 might be used as potential references for improving starter cultures, it should be considering the impact of the other co-existed species and the relative abundance, which could be delineated by meta-genomics analysis on the bacteria community in natural fermented dairy foods.

Xinjiang appears to have a particular richness of subsp. *bulgaricus* strains, as all five CCs were found there, and CC2–5 were exclusively isolated from this region. It was reported recently that the oldest known pieces of cheese were discovered in the tombs of an early Bronze Age cemetery in Xinjiang^23^,^24^. Accordingly, the production of fermented foods in Xinjiang can be traced back at least 3800 years. Xinjiang is also an important hub that has bridged human commercial activities between Eastern and Western societies for several thousands of years. Ancient traders may have brought fermented dairy products, and hence subsp. *bulgaricus* strains, from distant regions to Xinjiang, which promoted the spread and enriched the population diversity of the strains in this region. Therefore, the long history of fermented dairy food production, along with ancient human activity, might contribute to the higher number of subsp. *bulgaricus* CCs in Xinjiang, China. Comparatively, only 31 isolates consisting of 28 singletons and no clonal complexes were identified in Tibet. This restriction of clonal expansion might be attributed to the harsh environment of the plateau, which would suppress the growth and transmission of subsp. *bulgaricus* strains. Therefore, it would be interesting to explore the mechanisms of adaptive evolution of subsp. *bulgaricus* in different regions by deciphering the association between specific genomic features and environmental factors in their sampling regions.

## Conclusion

In this study, we designed an eight-locus MLST scheme and identified 251 subsp. *bulgaricus* isolates from a collection of 298 *L. delbrueckii* strains. In total, 106 STs, five CCs, and six lineages were identified amongst the subsp. *bulgaricus* strains, with different recombination rates and geographical distribution patterns observed across the lineages. This study improved our understanding of the genetic diversity of this important industrial bacterium. Further work based on whole genome sequence analysis would provide more subtle evolutionary details on subsp. *bulgaricus*, and provide insight into genetic mechanisms involved in the natural fermentation of dairy products. Together, this information will assist in improving bacterial starter cultures used in industrial production of dairy products.

## Materials and Methods

### Bacterial strains and DNA extraction

A total of 298 *L. delbrueckii* strains were analyzed in this study, including six type strains. Except for the *L. delbrueckii* subsp. *sunkii* type strain DSM24966^T^ (obtained from the Deutsche Sammlung von Mikroorganismen und Zellkulturen), completed whole genome sequences are available from the NCBI website (http://www.ncbi.nlm.nih.gov/genome/genomes/514) for all type strains, as well as for five other subspecies of *L. delbrueckii*. Details are listed in Supplementary Table S2. The strains included 284 isolates from the Collection Centre of Lactic Acid Bacteria (LABCC) of the Inner Mongolia Agriculture University, China, and three isolates derived from the National Bank for Industrial Microorganisms and Cell Cultures (NBIMCC) in Bulgaria. The study included strains isolated from naturally fermented dairy products made by nomads in the autonomous regions of Xinjiang and Tibet, and from the Qinghai, Sichuan, and Gansu provinces of China (the latter three provinces were adjacent to each other and designated “QSG of China” in this paper). Strains were also isolated from 13 provinces in Mongolia, and from Respublika Buryatiya, Kalmykia, and Tuwa, Russia (Supplementary Table S2), from 2005–2013. The three NBIMCC strains were isolated from yogurt starters used in Bulgaria and Denmark. The 284 LABCC strains were analyzed by 16S rRNA gene sequencing and identified as *L. delbrueckii* species. Detailed strain information is provided in Supplementary Table S2.

All 287 *L. delbrueckii* strains (LABCC: 284, NBIMCC: 3), as well as DSM24966^T^, were maintained in MRS broth (OXOID, Germany) at 37 °C for 18–24 h. Cells were harvested by centrifugation at 3000 rpm for 5 min, and cell pellets were used for total genomic DNA extraction as described previously^25^. Purified DNA was diluted to a final concentration of 100 ng/μL for further applications.

### Selection of MLST loci and primer design

Selection of the target housekeeping genes was based on the presence of SNPs between *L. delbrueckii* subsp. *bulgaricus* ATCC 11842^T^ (GenBank accession no. NC_008054) and *L. delbrueckii* ND02 (NC_014727) (Data not shown). We initially selected 12 loci for the MLST scheme, but four loci were abandoned because of amplification difficulties or low sequence quality. As a result, eight housekeeping genes encoding the following proteins were chosen for analysis: ATP-dependent protease ATP-binding subunit ClpX (*clpX*), chromosomal replication initiation protein (*dnaA*), CTP synthetase (*pyrG*), chaperonin GroEL (*groEL*), UDP-N-acetylmuramoylalanyl-D-glutamate-L-lysine ligase (*murE*), phenylalanyl-tRNA synthetase subunit alpha (*pheS*), recombinase A (*recA*), and DNA-directed RNA polymerase subunit beta (*rpoB*). All loci were present as a single copy, shared a conserved sequence, were widely distributed across the chromosome, and were mutually unlinked in location^26^. Primers to amplify the eight loci were designed using Primer Premier 5.0 (PREMIER Biosoft) based on the known genome sequence of *L. delbrueckii* subsp. *bulgaricus* ATCC 11842 ^T^. Primer sequences are listed in Supplementary Table S1.

### PCR amplification and sequencing

Genomic DNA from each strain was used as a template for amplification of MLST loci using a Veriti 96-Well Thermal Cycler (Applied Biosystems). For each target, a 50-μL PCR mixture was prepared, containing 150 ng of genomic DNA, 10 mM of each dNTP, 10 pmol of each primer, 2.5 U of *Taq* polymerase, and 1 × PCR buffer (with Mg^2+^). Parameters for the thermal cycler were: 94 °C for 4 min; 30 cycles of 94 °C for 1 min, 55–60 °C (optimal annealing temperatures for each locus are listed in Supplementary Table S1) for 45 s, and 72 °C for 1 min; and a final extension of 72 °C for 7 min. PCR products were electrophoresed in a 1.0% agarose gel. PCR products were sequenced by the Shanghai Majorbio Bio-pharm Technology Corporation using the same primers as for PCR.

### Diversity analyses

Both forward and reverse sequences for each strain were trimmed, aligned, and analyzed using BioNumerics software (version 6.6, Applied Maths). Allele definition was also performed with it. For each MLST locus, the sequences obtained for all isolates were compared, and each unique sequence was assigned with an allele number. Unique sequences were defined as those differing from the others at one or more nucleotides. Each isolate was unambiguously defined by an allele profile or sequence type (ST) derived from the combination of numbers corresponding to the alleles at each of the loci analyzed. The same ST was assigned to multiple strains when they shared the same allelic profile. Allelic profiles were used for subsequent analysis.

The mean GC content of the DNA along with the *d*_*N*_/*d*_*S*_ ratios (where *d*_*S*_ is the number of synonymous substitutions per synonymous site, and *d*_*N*_ is the number of nonsynonymous substitutions per nonsynonymous site) were calculated using START v2.0^27^. Nucleotide diversity per site (π) was estimated using DnaSP version 5.0^28^.

### Recombination analyses

The *phi*-test for recombination, based on individual loci from the whole strain collection, was performed using SplitsTree v4.12.6^18^. The P-value (a value of P < 0.05 was considered significant herein) indicated the DNA regions exhibiting the strongest evidence of mosaicism. The LDhat program^29^,^30^ of the RDP4 program^31^ was used to calculate per-site ρ/θ ratios based on concatenated sequences of the eight loci with 1,000,000 Markov Chain Monte Carlo (MCMC) updates. The parameters ρ and θ represented the rates of recombination and mutation respectively. The standardized index of association (*I*_*A*_^*S*^) was calculated using the lian program v3.6^19^.

### Phylogenetic analyses

Phylogenetic trees were constructed from the concatenated sequences (4261 bp) using the maximum likelihood method and the HKY97 model in PhyML^32^ with 1000 replications.

### Population structure analyses

eBURST^33^ was used to analyze population structure. STs were clustered into CCs based on the similarity of their allelic profiles. According to the grouping criterion, only one of the eight loci analyzed (SLVs) was allowed for intra-CC variation. The split decomposition method was used to assess the degree of tree-like structure for alleles of each locus and all STs using SplitsTree v4.12.6^18^,^34^. According to the algorithm, a tree-like structure indicates a clonal descent, while an interconnecting network or a parallelogram indicates recombination. Structure v2.3 with linkage model^35^ was used to infer the lineage ancestry of the unique STs, assuming that the ancestry of each ST was derived from K ancestral subpopulations. Three individual runs per value of K (chosen between 3 and 20) were performed using 500,000 MCMC iterations, consisting of 200,000 burn-in iterations and 300,000 sampling iterations. The K-value that generated the highest median posterior probability was used as the probable number of ancestral populations.

### Nucleotide sequence accession numbers

The sequences of the eight MLST loci have been deposited in the GenBank database under accession numbers KM227013–KM229308 and KP057215–KP057222.

## Additional Information

**How to cite this article**: Song, Y. *et al*. Genetic diversity and population structure of *Lactobacillus delbrueckii* subspecies *bulgaricus* isolated from naturally fermented dairy foods. *Sci. Rep.* **6**, 22704; doi: 10.1038/srep22704 (2016).

## Supplementary Material

Supplementary Information

## Figures and Tables

**Table 1:** Nucleotide and allele diversity of the MLST loci.

**Locus**	**Size (bp)**	**No. of**				***phi*****-test**	**G + C content (%)**	**π (nucleotide diversity)**	***d***_***N***_**/*****d***_***S***_
		**Alleles**	**polymorphic sites**	**nSNPs**	**sSNPs**				
*clpX*	508	21(13)	25(12)	8(5)	17(7)	0.84(0.23)	50.67(50.71)	0.0109(0.0065)	0.1542(0.2968)
*dnaA*	598	20(12)	28(9)	6(3)	22(6)	0.43(0.89)	52.85(52.72)	0.0099(0.0038)	0.0655(0.1869)
*groEL*	470	27(20)	27(17)	4(2)	23(15)	0.89(0.98)	50.76(50.69)	0.0081(0.0058)	0.0690(0.0630)
*murE*	477	22(14)	21(10)	6(1)	15(9)	0.81(0.02)	57.33(57.13)	0.0075(0.0048)	0.1547(0.0959)
*pheS*	491	22(15)	29(16)	4(3)	25(13)	0.86(1.00)	56.09(55.95)	0.0085(0.0062)	0.0287(0.0461)
*pyrG*	563	27(19)	37(19)	11(7)	26(12)	0.81(0.87)	54.32(54.27)	0.0090(0.0069)	0.1459(0.2401)
*recA*	551	25(16)	33(17)	0(0)	33(17)	0.96(1.00)	62.25(62.17)	0.0079(0.0050)	0.0000(0.0000)
*rpoB*	603	21(13)	24(13)	4(3)	20(10)	1.00(1.00)	54.16(54.14)	0.0056(0.0045)	0.0637(0.0924)
concatenated	4261	119(106)	224(113)	43(24)	181(89)	<0.00(<0.01)	54.8(54.8)	0.0042(0.0030)	0.0661(0.0661)

Note: the values indicate findings for *L. delbrueckii*, with subsp. *bulgaricus* values in parentheses.

**Table 2:** Recombination test and estimation results.

**Population (n)**	***phi*****-test**	**Recombination**				**Linkage disequilibrium**		
		**θ/site**	**ρ/site**	**LB 95%**	**UB 95%**	**ρ/θ**	**I**_**A**_^**S**^	**P value**
All (106ST)	0.0018	4.97E-03	2.33E-02	1.77E-02	3.06E-02	4.691	0.1325	<0.0001
L1 (26ST)	0.1296	2.94E-03	1.13E-02	7.95E-03	1.69E-02	3.841	0.0629	1.00E-03
L2 (6ST)	1	1.97E-03	9.83E-04	2.58E-04	2.59E-03	0.4987	0.1741	1.10E-02
L3 (14ST)	0.0308	2.10E-03	8.40E-02	2.69E-02	3.73E-01	39.969	0.0107	3.18E-01
L4 (10ST)	0.2819	2.03E-03	4.78E-03	7.69E-04	1.33E-02	2.356	0.016	3.45E-01
L5 (21ST)	0.1432	1.34E-03	1.26E-02	5.17E-03	3.13E-02	9.47	0.0973	<0.0001
L6 (29ST)	0.0048	2.00E-03	3.51E-02	1.20E-02	1.23E-01	17.587	−0.0178	7.51E-01
