# Supplementary material for: Genetic diversity and population structure of Lactobacillus delbrueckii subspecies bulgaricus isolated from naturally fermented dairy foods
Source: Sci Rep. 2016 Mar 4;6:22704. doi: 10.1038/srep22704 (PMC4778129; doi:10.1038/srep22704)
Supplement: Supplementary Information [file srep22704-s1.doc]

**Genetic diversity and population structure of *Lactobacillus* *delbrueckii* subspecies *bulgaricus* isolated from naturally fermented dairy foods**

Yuqin Song^1, +^, Zhihong Sun^1, +^, Chenyi Guo^2^, Yarong Wu^2^, Wenjun Liu^1^, Jie Yu^1^, Bilige Menghe^1^, Ruifu Yang^2^, Heping Zhang^1,*^

^1^Key Laboratory of Dairy Biotechnology and Engineering, Education Ministry of China, Inner Mongolia Agricultural University, Hohhot, Inner Mongolia 010018, China

^2^State Key Laboratory of Pathogen and Biosecurity, Beijing Institute of Microbiology and Epidemiology, Beijing 100071, China

^*^Corresponding author: hepingdd@vip.sina.com

^+^These authors contributed equally to this work

Supplementary materials

**Supplementary Figure S1.** **Maximum-likelihood tree of *L. delbrueckii*.** The maximum likelihood tree for 298 *L. delbrueckii* isolates was constructed using PhyML based on the concatenated sequences of eight MLST loci. *L. equicursoris* ATCC19284^T^ (accession no.: NZ_CAMA00000000) was used as an outgroup, and six type strains (indicated by bold font and with a superscript “^T^” after the strain name) were included as indicators of the subspecies. Bootstrap values are indicated for all branches.

**Supplementary Figure S2. Neighbor-joining tree constructed based on sequences for individual loci.**

**Supplementary Figure S3.** **Geographical distribution of subsp*. bulgaricus* strains in this research.** The map was performed in R 3.1.1 (<http://www.r-project.org/>) using the maps (Minka and Deckmyn, 2015) package. The size of the circles indicates the number of strains isolated from the corresponding region, and the colors represent the clonal complexes, singletons, and doubletons. The isolation regions include the Xinjiang and Tibet autonomous regions, and Qinghai, Sichuan, and Gansu provinces, China (the three provinces were named QSG in this paper), as well as Mongolia and Russia.

| **Supplementary Table S1. Primers used for amplification and sequencing** | | | | | | |
| --- | --- | --- | --- | --- | --- | --- |
| Gene | Primer Name | Sequence (5'→3') | Annealing Temperature (℃) | Amplification product position on the gene | Gene position on genome^a^ | Template size (nt) |
| clpX | B_clpX_primerF | AAGTCTGCCAGCCAAGTAA | 60 | 52-736 | 670313-671566 | 685 |
|  | B_clpX_primerR | CGCCGACAATGAAGAGGAT |  |  |  |  |
| dnaA | B_dnaA_primerF | GAGCCCAGCAGCGAAAG | 60 | 259-1033 | 323-1687 | 775 |
|  | B_dnaA_primerR | CTAAGCCAATGTTGATGTCC |  |  |  |  |
| groEL | B_groEL_primerF | TCGGCAAGGACGGTGTT | 57 | 500-1134 | 1392354-1393967 | 635 |
|  | B_groEL_primerR | GTGGATTACGGCTACGC |  |  |  |  |
| murE | B_murE_primerF | CTGAGCAGCCATACCCG | 55 | 215-922 | 1710614-1712164 | 708 |
|  | B_murE_primerR | CCTTGCTTGAAGCCGTAG |  |  |  |  |
| pheS | B_pheS_primerF | CATCGGCATGAGCTACCA | 55 | 369-1042 | 1283032-1284081 | 674 |
|  | B_pheS_primerR | CCTCCTGACGGAATTGTTG |  |  |  |  |
| pyrG | B_pyrG_primerF | AAGCCGACCCAGCAATC | 57 | 568-1309 | 301679-303298 | 742 |
|  | B_pyrG_primerR | AGCCCAGACGCAAGGTG |  |  |  |  |
| recA | B_recA_primerF | ATGCGGATGGGCGAGAA | 59 | 79-772 | 507700-508695 | 694 |
|  | B_recA_primerR | CTACCTTAAATGGCGGAGC |  |  |  |  |
| rpoB | B_rpoB_primerF | GGCGGAAAGAGTTATCGT | 58 | 399-1170 | 334519-338184 | 772 |
|  | B_rpoB_primerR | GATGTCGGCTGGAGTGAT |  |  |  |  |
| Note: ^a^: The genome refers to ATCC11842^T^ (Accession No: NC_008054). | | | | |  |  |

| **Supplementary Table S2**. Background information on *L. delbrueckii* strains used in this study. | | | | | | | | | | | | | | | | | | | | | |
| --- | --- | --- | --- | --- | --- | --- | --- | --- | --- | --- | --- | --- | --- | --- | --- | --- | --- | --- | --- | --- | --- |
| Strains | Regions | Source | Year | Assession No. of 16S rRNA | Allele Profiles | | | | | | | | | | ST | | Cluster | | Lineage | | MLST.ssp |
|  |  |  |  |  | *clpX* | *dnaA* | *groEL* | *murE* | *pheS* | *pyrG* | *recA* | | *rpoB* | |  |  |  |  |  |  |  |
| IMAU20102 | Mongolia | Fermented camel milk | 2005 | FJ845002 | 4 | 4 | 3 | 1 | 3 | 5 | 1 | | 4 | | ST7 | | CC1 | | L6 | | bulgaricus |
| IMAU20180 | Mongolia | Fermented cow milk | 2009 | HM057917 | 4 | 4 | 3 | 1 | 3 | 5 | 1 | | 5 | | ST10 | | CC1 | | L6 | | bulgaricus |
| IMAU20327 | Mongolia | Fermented cow milk | 2009 | HM058057 | 4 | 4 | 3 | 1 | 3 | 5 | 1 | | 5 | | ST10 | | CC1 | | L6 | | bulgaricus |
| IMAU20329 | Mongolia | Fermented cow milk | 2009 | HM058059 | 4 | 4 | 3 | 1 | 3 | 5 | 1 | | 5 | | ST10 | | CC1 | | L6 | | bulgaricus |
| IMAU20331 | Mongolia | Fermented cow milk | 2009 | HM058061 | 4 | 4 | 3 | 1 | 3 | 5 | 1 | | 5 | | ST10 | | CC1 | | L6 | | bulgaricus |
| IMAU20382 | Mongolia | Fermented cow milk | 2009 | HM058107 | 4 | 4 | 3 | 1 | 3 | 5 | 1 | | 5 | | ST10 | | CC1 | | L6 | | bulgaricus |
| IMAU20383 | Mongolia | Fermented cow milk | 2009 | HM058108 | 4 | 4 | 3 | 1 | 3 | 5 | 1 | | 5 | | ST10 | | CC1 | | L6 | | bulgaricus |
| IMAU20429 | Mongolia | Fermented cow milk | 2009 | HM058153 | 4 | 4 | 3 | 1 | 3 | 5 | 1 | | 5 | | ST10 | | CC1 | | L6 | | bulgaricus |
| IMAU20670 | Mongolia | Fermented cow milk | 2009 | HM058382 | 4 | 4 | 3 | 1 | 3 | 5 | 1 | | 5 | | ST10 | | CC1 | | L6 | | bulgaricus |
| IMAU20708 | Mongolia | Fermented cow milk | 2009 | HM058420 | 4 | 4 | 3 | 1 | 3 | 5 | 1 | | 5 | | ST10 | | CC1 | | L6 | | bulgaricus |
| IMAU20709 | Mongolia | Fermented cow milk | 2009 | HM058421 | 4 | 4 | 3 | 1 | 3 | 5 | 1 | | 5 | | ST10 | | CC1 | | L6 | | bulgaricus |
| IMAU20724 | Mongolia | Fermented cow milk | 2009 | HM058435 | 4 | 4 | 3 | 1 | 3 | 5 | 1 | | 5 | | ST10 | | CC1 | | L6 | | bulgaricus |
| IMAU20753 | Mongolia | Fermented cow milk | 2009 | HM058463 | 4 | 4 | 3 | 1 | 3 | 5 | 1 | | 5 | | ST10 | | CC1 | | L6 | | bulgaricus |
| IMAU20754 | Mongolia | Fermented cow milk | 2009 | HM058464 | 4 | 4 | 3 | 1 | 3 | 5 | 1 | | 5 | | ST10 | | CC1 | | L6 | | bulgaricus |
| IMAU20755 | Mongolia | Fermented cow milk | 2009 | HM058465 | 4 | 4 | 3 | 1 | 3 | 5 | 1 | | 5 | | ST10 | | CC1 | | L6 | | bulgaricus |
| IMAU20207 | Mongolia | Fermented cow milk | 2009 | HM057943 | 4 | 4 | 3 | 1 | 3 | 5 | 5 | | 5 | | ST12 | | CC1 | | L6 | | bulgaricus |
| IMAU20360 | Mongolia | Fermented cow milk | 2009 | HM058088 | 4 | 4 | 3 | 1 | 3 | 5 | 5 | | 5 | | ST12 | | CC1 | | L6 | | bulgaricus |
| IMAU20425 | Mongolia | Fermented cow milk | 2009 | HM058149 | 4 | 4 | 3 | 1 | 3 | 5 | 5 | | 5 | | ST12 | | CC1 | | L6 | | bulgaricus |
| IMAU20761 | Mongolia | Fermented cow milk | 2009 | HM058471 | 4 | 4 | 3 | 1 | 3 | 5 | 5 | | 5 | | ST12 | | CC1 | | L6 | | bulgaricus |
| IMAU20762 | Mongolia | Fermented cow milk | 2009 | HM058472 | 4 | 4 | 3 | 1 | 3 | 5 | 5 | | 5 | | ST12 | | CC1 | | L6 | | bulgaricus |
| IMAU20770 | Mongolia | Fermented cow milk | 2009 | HM058480 | 4 | 4 | 3 | 1 | 3 | 5 | 5 | | 5 | | ST12 | | CC1 | | L6 | | bulgaricus |
| IMAU20775 | Mongolia | Fermented cow milk | 2009 | HM058485 | 4 | 4 | 3 | 1 | 3 | 5 | 5 | | 5 | | ST12 | | CC1 | | L6 | | bulgaricus |
| IMAU20783 | Mongolia | Fermented cow milk | 2009 | HM058493 | 4 | 4 | 3 | 1 | 3 | 5 | 5 | | 5 | | ST12 | | CC1 | | L6 | | bulgaricus |
| IMAU20210 | Mongolia | Fermented cow milk | 2009 | HM057946 | 4 | 4 | 3 | 1 | 3 | 5 | 1 | | 6 | | ST13 | | CC1 | | L6 | | bulgaricus |
| IMAU20215 | Mongolia | Fermented cow milk | 2009 | HM057950 | 4 | 4 | 3 | 1 | 3 | 5 | 1 | | 6 | | ST13 | | CC1 | | L6 | | bulgaricus |
| IMAU20216 | Mongolia | Fermented cow milk | 2009 | HM057951 | 4 | 4 | 3 | 1 | 3 | 5 | 1 | | 6 | | ST13 | | CC1 | | L6 | | bulgaricus |
| IMAU20217 | Mongolia | Fermented cow milk | 2009 | HM057952 | 4 | 4 | 3 | 1 | 3 | 5 | 1 | | 6 | | ST13 | | CC1 | | L6 | | bulgaricus |
| IMAU20220 | Mongolia | Fermented cow milk | 2009 | HM057954 | 4 | 4 | 3 | 1 | 3 | 5 | 1 | | 6 | | ST13 | | CC1 | | L6 | | bulgaricus |
| IMAU20221 | Mongolia | Fermented cow milk | 2009 | HM057955 | 4 | 4 | 3 | 1 | 3 | 5 | 1 | | 6 | | ST13 | | CC1 | | L6 | | bulgaricus |
| IMAU20222 | Mongolia | Fermented cow milk | 2009 | HM057956 | 4 | 4 | 3 | 1 | 3 | 5 | 1 | | 6 | | ST13 | | CC1 | | L6 | | bulgaricus |
| IMAU20227 | Mongolia | Fermented cow milk | 2009 | HM057961 | 4 | 4 | 3 | 1 | 3 | 5 | 1 | | 6 | | ST13 | | CC1 | | L6 | | bulgaricus |
| IMAU20228 | Mongolia | Fermented cow milk | 2009 | HM057962 | 4 | 4 | 3 | 1 | 3 | 5 | 1 | | 6 | | ST13 | | CC1 | | L6 | | bulgaricus |
| IMAU20234 | Mongolia | Fermented cow milk | 2009 | HM057968 | 4 | 4 | 3 | 1 | 3 | 5 | 1 | | 6 | | ST13 | | CC1 | | L6 | | bulgaricus |
| IMAU20295 | Mongolia | Fermented cow milk | 2009 | HM058026 | 4 | 4 | 3 | 1 | 3 | 5 | 1 | | 6 | | ST13 | | CC1 | | L6 | | bulgaricus |
| IMAU20379 | Mongolia | Fermented cow milk | 2009 | HM058104 | 4 | 4 | 3 | 1 | 3 | 5 | 1 | | 6 | | ST13 | | CC1 | | L6 | | bulgaricus |
| IMAU20396 | Mongolia | Fermented cow milk | 2009 | HM058120 | 4 | 4 | 3 | 1 | 3 | 5 | 1 | | 6 | | ST13 | | CC1 | | L6 | | bulgaricus |
| IMAU20401 | Mongolia | Fermented cow milk | 2009 | HM058125 | 4 | 4 | 3 | 1 | 3 | 5 | 1 | | 6 | | ST13 | | CC1 | | L6 | | bulgaricus |
| IMAU20402 | Mongolia | Fermented cow milk | 2009 | HM058126 | 4 | 4 | 3 | 1 | 3 | 5 | 1 | | 6 | | ST13 | | CC1 | | L6 | | bulgaricus |
| IMAU20404 | Mongolia | Fermented cow milk | 2009 | HM058128 | 4 | 4 | 3 | 1 | 3 | 5 | 1 | | 6 | | ST13 | | CC1 | | L6 | | bulgaricus |
| IMAU20410 | Mongolia | Fermented cow milk | 2009 | HM058134 | 4 | 4 | 3 | 1 | 3 | 5 | 1 | | 6 | | ST13 | | CC1 | | L6 | | bulgaricus |
| IMAU20422 | Mongolia | Fermented cow milk | 2009 | HM058146 | 4 | 4 | 3 | 1 | 3 | 5 | 1 | | 6 | | ST13 | | CC1 | | L6 | | bulgaricus |
| IMAU20214 | Mongolia | Fermented cow milk | 2009 | HM057949 | 4 | 4 | 3 | 1 | 3 | 5 | 1 | | 3 | | ST14 | | CC1 | | L6 | | bulgaricus |
| IMAU20290 | Mongolia | Fermented cow milk | 2009 | HM058021 | 4 | 4 | 3 | 1 | 7 | 5 | 1 | | 4 | | ST16 | | CC1 | | L6 | | bulgaricus |
| IMAU20421 | Mongolia | Fermented cow milk | 2009 | HM058145 | 4 | 4 | 3 | 7 | 3 | 5 | 1 | | 5 | | ST18 | | CC1 | | L6 | | bulgaricus |
| IMAU20763 | Mongolia | Fermented cow milk | 2009 | HM058473 | 4 | 4 | 3 | 7 | 3 | 5 | 1 | | 5 | | ST18 | | CC1 | | L6 | | bulgaricus |
| IMAU20790 | Mongolia | Fermented cow milk | 2009 | HM058500 | 4 | 4 | 3 | 7 | 3 | 5 | 1 | | 5 | | ST18 | | CC1 | | L6 | | bulgaricus |
| IMAU20355 | Mongolia | Fermented cow milk | 2009 | HM058083 | 4 | 4 | 3 | 1 | 9 | 5 | 1 | | 5 | | ST19 | | CC1 | | L6 | | bulgaricus |
| IMAU20364 | Mongolia | Fermented cow milk | 2009 | HM058092 | 4 | 4 | 3 | 8 | 3 | 5 | 1 | | 5 | | ST20 | | CC1 | | L6 | | bulgaricus |
| IMAU20366 | Mongolia | Fermented cow milk | 2009 | HM058094 | 4 | 4 | 3 | 1 | 3 | 10 | 1 | | 5 | | ST21 | | CC1 | | L6 | | bulgaricus |
| IMAU20403 | Mongolia | Fermented cow milk | 2009 | HM058127 | 4 | 4 | 3 | 1 | 3 | 5 | 5 | | 6 | | ST22 | | CC1 | | L6 | | bulgaricus |
| IMAU20426 | Mongolia | Fermented cow milk | 2009 | HM058150 | 4 | 4 | 3 | 1 | 9 | 5 | 1 | | 6 | | ST23 | | CC1 | | L6 | | bulgaricus |
| IMAU20428 | Mongolia | Fermented cow milk | 2009 | HM058152 | 8 | 4 | 3 | 7 | 3 | 5 | 1 | | 5 | | ST24 | | CC1 | | L6 | | bulgaricus |
| IMAU20499 | Mongolia | Fermented cow milk | 2009 | HM058218 | 4 | 4 | 3 | 1 | 3 | 5 | 5 | | 4 | | ST27 | | CC1 | | L6 | | bulgaricus |
| IMAU20598 | Mongolia | Fermented cow milk | 2009 | HM058314 | 4 | 4 | 3 | 1 | 3 | 5 | 4 | | 4 | | ST31 | | CC1 | | L6 | | bulgaricus |
| IMAU20792 | Mongolia | Fermented cow milk | 2009 | HM058502 | 8 | 4 | 3 | 1 | 3 | 5 | 1 | | 4 | | ST35 | | CC1 | | L6 | | bulgaricus |
| IMAU20133 | Mongolia | Fermented cow milk | 2009 | HM057871 | 4 | 4 | 3 | 1 | 3 | 5 | 1 | | 4 | | ST7 | | CC1 | | L6 | | bulgaricus |
| IMAU20136 | Mongolia | Fermented cow milk | 2009 | HM057874 | 4 | 4 | 3 | 1 | 3 | 5 | 1 | | 4 | | ST7 | | CC1 | | L6 | | bulgaricus |
| IMAU20238 | Mongolia | Fermented cow milk | 2009 | HM057972 | 4 | 4 | 3 | 1 | 3 | 5 | 1 | | 4 | | ST7 | | CC1 | | L6 | | bulgaricus |
| IMAU20239 | Mongolia | Fermented cow milk | 2009 | HM057973 | 4 | 4 | 3 | 1 | 3 | 5 | 1 | | 4 | | ST7 | | CC1 | | L6 | | bulgaricus |
| IMAU20240 | Mongolia | Fermented cow milk | 2009 | HM057974 | 4 | 4 | 3 | 1 | 3 | 5 | 1 | | 4 | | ST7 | | CC1 | | L6 | | bulgaricus |
| IMAU20255 | Mongolia | Fermented cow milk | 2009 | HM057989 | 4 | 4 | 3 | 1 | 3 | 5 | 1 | | 4 | | ST7 | | CC1 | | L6 | | bulgaricus |
| IMAU20257 | Mongolia | Fermented cow milk | 2009 | HM057991 | 4 | 4 | 3 | 1 | 3 | 5 | 1 | | 4 | | ST7 | | CC1 | | L6 | | bulgaricus |
| IMAU20269 | Mongolia | Fermented cow milk | 2009 | HM058003 | 4 | 4 | 3 | 1 | 3 | 5 | 1 | | 4 | | ST7 | | CC1 | | L6 | | bulgaricus |
| IMAU20273 | Mongolia | Fermented cow milk | 2009 | HM058007 | 4 | 4 | 3 | 1 | 3 | 5 | 1 | | 4 | | ST7 | | CC1 | | L6 | | bulgaricus |
| IMAU20277 | Mongolia | Fermented cow milk | 2009 | HM058011 | 4 | 4 | 3 | 1 | 3 | 5 | 1 | | 4 | | ST7 | | CC1 | | L6 | | bulgaricus |
| IMAU20278 | Mongolia | Fermented cow milk | 2009 | HM058012 | 4 | 4 | 3 | 1 | 3 | 5 | 1 | | 4 | | ST7 | | CC1 | | L6 | | bulgaricus |
| IMAU20279 | Mongolia | Fermented cow milk | 2009 | HM058013 | 4 | 4 | 3 | 1 | 3 | 5 | 1 | | 4 | | ST7 | | CC1 | | L6 | | bulgaricus |
| IMAU20281 | Mongolia | Fermented cow milk | 2009 | HM058015 | 4 | 4 | 3 | 1 | 3 | 5 | 1 | | 4 | | ST7 | | CC1 | | L6 | | bulgaricus |
| IMAU20282 | Mongolia | Fermented cow milk | 2009 | HM058016 | 4 | 4 | 3 | 1 | 3 | 5 | 1 | | 4 | | ST7 | | CC1 | | L6 | | bulgaricus |
| IMAU20289 | Mongolia | Fermented cow milk | 2009 | HM058020 | 4 | 4 | 3 | 1 | 3 | 5 | 1 | | 4 | | ST7 | | CC1 | | L6 | | bulgaricus |
| IMAU20291 | Mongolia | Fermented cow milk | 2009 | HM058022 | 4 | 4 | 3 | 1 | 3 | 5 | 1 | | 4 | | ST7 | | CC1 | | L6 | | bulgaricus |
| IMAU20292 | Mongolia | Fermented cow milk | 2009 | HM058023 | 4 | 4 | 3 | 1 | 3 | 5 | 1 | | 4 | | ST7 | | CC1 | | L6 | | bulgaricus |
| IMAU20298 | Mongolia | Fermented cow milk | 2009 | HM058029 | 4 | 4 | 3 | 1 | 3 | 5 | 1 | | 4 | | ST7 | | CC1 | | L6 | | bulgaricus |
| IMAU20302 | Mongolia | Fermented cow milk | 2009 | HM058032 | 4 | 4 | 3 | 1 | 3 | 5 | 1 | | 4 | | ST7 | | CC1 | | L6 | | bulgaricus |
| IMAU20353 | Mongolia | Fermented cow milk | 2009 | HM058081 | 4 | 4 | 3 | 1 | 3 | 5 | 1 | | 4 | | ST7 | | CC1 | | L6 | | bulgaricus |
| IMAU20412 | Mongolia | Fermented cow milk | 2009 | HM058136 | 4 | 4 | 3 | 1 | 3 | 5 | 1 | | 4 | | ST7 | | CC1 | | L6 | | bulgaricus |
| IMAU20423 | Mongolia | Fermented cow milk | 2009 | HM058147 | 4 | 4 | 3 | 1 | 3 | 5 | 1 | | 4 | | ST7 | | CC1 | | L6 | | bulgaricus |
| IMAU20427 | Mongolia | Fermented cow milk | 2009 | HM058151 | 4 | 4 | 3 | 1 | 3 | 5 | 1 | | 4 | | ST7 | | CC1 | | L6 | | bulgaricus |
| IMAU20553 | Mongolia | Fermented cow milk | 2009 | HM058272 | 4 | 4 | 3 | 1 | 3 | 5 | 1 | | 4 | | ST7 | | CC1 | | L6 | | bulgaricus |
| IMAU205631 | Mongolia | Fermented cow milk | 2009 | HM058345 | 4 | 4 | 3 | 1 | 3 | 5 | 1 | | 4 | | ST7 | | CC1 | | L6 | | bulgaricus |
| IMAU20571 | Mongolia | Fermented cow milk | 2009 | HM058289 | 4 | 4 | 3 | 1 | 3 | 5 | 1 | | 4 | | ST7 | | CC1 | | L6 | | bulgaricus |
| IMAU20632 | Mongolia | Fermented cow milk | 2009 | HM058346 | 4 | 4 | 3 | 1 | 3 | 5 | 1 | | 4 | | ST7 | | CC1 | | L6 | | bulgaricus |
| IMAU20635 | Mongolia | Fermented cow milk | 2009 | HM058349 | 4 | 4 | 3 | 1 | 3 | 5 | 1 | | 4 | | ST7 | | CC1 | | L6 | | bulgaricus |
| IMAU20679 | Mongolia | Fermented cow milk | 2009 | HM058391 | 4 | 4 | 3 | 1 | 3 | 5 | 1 | | 4 | | ST7 | | CC1 | | L6 | | bulgaricus |
| IMAU20745 | Mongolia | Fermented cow milk | 2009 | HM058456 | 4 | 4 | 3 | 1 | 3 | 5 | 1 | | 4 | | ST7 | | CC1 | | L6 | | bulgaricus |
| IMAU20746 | Mongolia | Fermented cow milk | 2009 | HM218014 | 4 | 4 | 3 | 1 | 3 | 5 | 1 | | 4 | | ST7 | | CC1 | | L6 | | bulgaricus |
| IMAU20748 | Mongolia | Fermented cow milk | 2009 | HM058458 | 4 | 4 | 3 | 1 | 3 | 5 | 1 | | 4 | | ST7 | | CC1 | | L6 | | bulgaricus |
| IMAU20758 | Mongolia | Fermented cow milk | 2009 | HM058468 | 4 | 4 | 3 | 1 | 3 | 5 | 1 | | 4 | | ST7 | | CC1 | | L6 | | bulgaricus |
| IMAU20769 | Mongolia | Fermented cow milk | 2009 | HM058479 | 4 | 4 | 3 | 1 | 3 | 5 | 1 | | 4 | | ST7 | | CC1 | | L6 | | bulgaricus |
| IMAU20776 | Mongolia | Fermented cow milk | 2009 | HM058486 | 4 | 4 | 3 | 1 | 3 | 5 | 1 | | 4 | | ST7 | | CC1 | | L6 | | bulgaricus |
| IMAU20777 | Mongolia | Fermented cow milk | 2009 | HM058487 | 4 | 4 | 3 | 1 | 3 | 5 | 1 | | 4 | | ST7 | | CC1 | | L6 | | bulgaricus |
| IMAU20788 | Mongolia | Fermented cow milk | 2009 | HM058498 | 4 | 4 | 3 | 1 | 3 | 5 | 1 | | 4 | | ST7 | | CC1 | | L6 | | bulgaricus |
| IMAU20794 | Mongolia | Fermented cow milk | 2009 | HM058504 | 4 | 4 | 3 | 1 | 3 | 5 | 1 | | 4 | | ST7 | | CC1 | | L6 | | bulgaricus |
| IMAU20334 | Mongolia | Fermented mare milk | 2009 | HM058064 | 4 | 4 | 3 | 1 | 3 | 5 | 1 | | 5 | | ST10 | | CC1 | | L6 | | bulgaricus |
| IMAU20335 | Mongolia | Fermented mare milk | 2009 | HM058514 | 4 | 4 | 3 | 1 | 3 | 5 | 1 | | 5 | | ST10 | | CC1 | | L6 | | bulgaricus |
| IMAU20339 | Mongolia | Fermented mare milk | 2009 | HM058068 | 4 | 4 | 3 | 1 | 3 | 5 | 5 | | 5 | | ST12 | | CC1 | | L6 | | bulgaricus |
| IMAU20342 | Mongolia | Fermented mare milk | 2009 | HM058071 | 4 | 4 | 3 | 7 | 3 | 5 | 1 | | 5 | | ST18 | | CC1 | | L6 | | bulgaricus |
| IMAU20337 | Mongolia | Fermented mare milk | 2009 | HM058066 | 4 | 4 | 3 | 1 | 3 | 5 | 1 | | 4 | | ST7 | | CC1 | | L6 | | bulgaricus |
| IMAU20341 | Mongolia | Fermented mare milk | 2009 | HM058070 | 4 | 4 | 3 | 1 | 3 | 5 | 1 | | 4 | | ST7 | | CC1 | | L6 | | bulgaricus |
| IMAU20452 | Mongolia | Fermented yak milk | 2009 | HM058175 | 4 | 4 | 3 | 1 | 3 | 5 | 5 | | 5 | | ST12 | | CC1 | | L6 | | bulgaricus |
| IMAU20450 | Mongolia | Fermented yak milk | 2009 | HM218005 | 4 | 4 | 3 | 1 | 3 | 5 | 1 | | 6 | | ST13 | | CC1 | | L6 | | bulgaricus |
| IMAU90010 | Russia | Fermented cow milk | 2012 | KC836528 | 4 | 4 | 3 | 1 | 14 | 5 | 5 | | 4 | | ST116 | | CC1 | | L6 | | bulgaricus |
| IMAU95016 | Russia | Fermented cow milk | 2013 | KJ026576 | 4 | 4 | 3 | 1 | 3 | 5 | 5 | | 5 | | ST12 | | CC1 | | L6 | | bulgaricus |
| IMAU95020 | Russia | Fermented cow milk | 2013 | KJ026580 | 4 | 4 | 3 | 1 | 3 | 5 | 5 | | 5 | | ST12 | | CC1 | | L6 | | bulgaricus |
| IMAU95037 | Russia | Fermented cow milk | 2013 | KJ026597 | 4 | 4 | 3 | 1 | 3 | 5 | 5 | | 5 | | ST12 | | CC1 | | L6 | | bulgaricus |
| IMAU95046 | Russia | Fermented cow milk | 2013 | KJ026606 | 4 | 4 | 3 | 1 | 3 | 5 | 5 | | 5 | | ST12 | | CC1 | | L6 | | bulgaricus |
| IMAU95052 | Russia | Fermented cow milk | 2013 | KJ026612 | 4 | 4 | 3 | 1 | 3 | 5 | 5 | | 5 | | ST12 | | CC1 | | L6 | | bulgaricus |
| IMAU95055 | Russia | Fermented cow milk | 2013 | KJ026615 | 4 | 4 | 3 | 1 | 3 | 5 | 5 | | 5 | | ST12 | | CC1 | | L6 | | bulgaricus |
| IMAU95057 | Russia | Fermented cow milk | 2013 | KJ026617 | 4 | 4 | 3 | 1 | 3 | 5 | 5 | | 5 | | ST12 | | CC1 | | L6 | | bulgaricus |
| IMAU95061 | Russia | Fermented cow milk | 2013 | KJ026621 | 4 | 4 | 3 | 1 | 3 | 5 | 5 | | 5 | | ST12 | | CC1 | | L6 | | bulgaricus |
| IMAU95087 | Russia | Fermented cow milk | 2013 | KJ026647 | 4 | 4 | 3 | 1 | 3 | 5 | 5 | | 5 | | ST12 | | CC1 | | L6 | | bulgaricus |
| IMAU95089 | Russia | Fermented cow milk | 2013 | KJ026649 | 4 | 4 | 3 | 1 | 3 | 5 | 5 | | 5 | | ST12 | | CC1 | | L6 | | bulgaricus |
| IMAU95095 | Russia | Fermented cow milk | 2013 | KJ026655 | 4 | 4 | 3 | 1 | 3 | 5 | 5 | | 5 | | ST12 | | CC1 | | L6 | | bulgaricus |
| IMAU95097 | Russia | Fermented cow milk | 2013 | KJ026657 | 4 | 4 | 3 | 1 | 3 | 5 | 5 | | 5 | | ST12 | | CC1 | | L6 | | bulgaricus |
| IMAU95098 | Russia | Fermented cow milk | 2013 | KJ026658 | 4 | 4 | 3 | 1 | 3 | 5 | 5 | | 5 | | ST12 | | CC1 | | L6 | | bulgaricus |
| IMAU95103 | Russia | Fermented cow milk | 2013 | KJ026663 | 4 | 4 | 3 | 1 | 3 | 5 | 5 | | 5 | | ST12 | | CC1 | | L6 | | bulgaricus |
| IMAU95106 | Russia | Fermented cow milk | 2013 | KJ026666 | 4 | 4 | 3 | 1 | 3 | 5 | 5 | | 5 | | ST12 | | CC1 | | L6 | | bulgaricus |
| IMAU95107 | Russia | Fermented cow milk | 2013 | KJ026667 | 4 | 4 | 3 | 1 | 3 | 5 | 5 | | 5 | | ST12 | | CC1 | | L6 | | bulgaricus |
| IMAU95110 | Russia | Fermented cow milk | 2013 | KJ026670 | 4 | 4 | 3 | 1 | 3 | 5 | 5 | | 5 | | ST12 | | CC1 | | L6 | | bulgaricus |
| IMAU95112 | Russia | Fermented cow milk | 2013 | KJ026672 | 4 | 4 | 3 | 1 | 3 | 5 | 5 | | 5 | | ST12 | | CC1 | | L6 | | bulgaricus |
| IMAU80396 | QSG of China^b^ | Qula | 2009 | HM058662 | 4 | 4 | 3 | 1 | 3 | 5 | 1 | | 6 | | ST13 | | CC1 | | L6 | | bulgaricus |
| IMAU32362 | Xinjiang, China | Fermented cow milk | 2012 | KF149040 | 4 | 4 | 3 | 1 | 3 | 4 | 1 | | 6 | | ST63 | | CC1 | | L6 | | bulgaricus |
| IMAU32379 | Xinjiang, China | Fermented cow milk | 2012 | KF149057 | 4 | 4 | 3 | 1 | 13 | 5 | 1 | | 6 | | ST66 | | CC1 | | L6 | | bulgaricus |
| IMAU32076 | Xinjiang, China | Fermented cow milk | 2012 | KF148764 | 9 | 9 | 3 | 1 | 3 | 15 | 10 | | 6 | | ST39 | | CC2 | | L5 | | bulgaricus |
| IMAU32087 | Xinjiang, China | Fermented cow milk | 2012 | KF148773 | 9 | 9 | 11 | 1 | 11 | 15 | 10 | | 6 | | ST41 | | CC2 | | L5 | | bulgaricus |
| IMAU32312 | Xinjiang, China | Fermented cow milk | 2012 | KF148990 | 9 | 9 | 11 | 1 | 11 | 15 | 10 | | 6 | | ST41 | | CC2 | | L5 | | bulgaricus |
| IMAU32318 | Xinjiang, China | Fermented cow milk | 2012 | KF148996 | 9 | 9 | 11 | 1 | 11 | 15 | 10 | | 6 | | ST41 | | CC2 | | L5 | | bulgaricus |
| IMAU32320 | Xinjiang, China | Fermented cow milk | 2012 | KF148998 | 9 | 9 | 11 | 1 | 11 | 15 | 10 | | 6 | | ST41 | | CC2 | | L5 | | bulgaricus |
| IMAU32326 | Xinjiang, China | Fermented cow milk | 2012 | KF149004 | 9 | 9 | 11 | 1 | 11 | 15 | 10 | | 6 | | ST41 | | CC2 | | L5 | | bulgaricus |
| IMAU32346 | Xinjiang, China | Fermented cow milk | 2012 | KF149024 | 9 | 9 | 11 | 1 | 11 | 15 | 10 | | 6 | | ST41 | | CC2 | | L5 | | bulgaricus |
| IMAU32349 | Xinjiang, China | Fermented cow milk | 2012 | KF149027 | 9 | 9 | 11 | 1 | 11 | 15 | 10 | | 6 | | ST41 | | CC2 | | L5 | | bulgaricus |
| IMAU32111 | Xinjiang, China | Fermented cow milk | 2012 | KF148796 | 9 | 9 | 11 | 1 | 3 | 15 | 10 | | 6 | | ST44 | | CC2 | | L5 | | bulgaricus |
| IMAU32223 | Xinjiang, China | Fermented cow milk | 2012 | KF148907 | 9 | 9 | 11 | 1 | 3 | 15 | 10 | | 6 | | ST44 | | CC2 | | L5 | | bulgaricus |
| IMAU32188 | Xinjiang, China | Fermented cow milk | 2012 | KF148872 | 4 | 9 | 11 | 1 | 11 | 15 | 10 | | 6 | | ST51 | | CC2 | | L5 | | bulgaricus |
| IMAU32190 | Xinjiang, China | Fermented cow milk | 2012 | KF148874 | 9 | 2 | 11 | 1 | 11 | 15 | 10 | | 6 | | ST52 | | CC2 | | L5 | | bulgaricus |
| IMAU32222 | Xinjiang, China | Fermented cow milk | 2012 | KF148906 | 9 | 9 | 13 | 1 | 11 | 15 | 10 | | 6 | | ST54 | | CC2 | | L5 | | bulgaricus |
| IMAU32265 | Xinjiang, China | Fermented cow milk | 2012 | KF148949 | 9 | 9 | 13 | 1 | 11 | 15 | 10 | | 6 | | ST54 | | CC2 | | L5 | | bulgaricus |
| IMAU32317 | Xinjiang, China | Fermented cow milk | 2012 | KF148995 | 9 | 4 | 13 | 1 | 11 | 15 | 10 | | 6 | | ST60 | | CC2 | | L5 | | bulgaricus |
| IMAU32341 | Xinjiang, China | Fermented cow milk | 2012 | KF149019 | 4 | 9 | 11 | 1 | 3 | 15 | 10 | | 6 | | ST61 | | CC2 | | L5 | | bulgaricus |
| IMAU32344 | Xinjiang, China | Fermented cow milk | 2012 | KF149022 | 4 | 9 | 11 | 1 | 3 | 15 | 10 | | 6 | | ST61 | | CC2 | | L5 | | bulgaricus |
| IMAU32687 | Xinjiang, China | Fermented cow milk | 2012 | KF149355 | 9 | 4 | 11 | 1 | 11 | 15 | 10 | | 6 | | ST68 | | CC2 | | L5 | | bulgaricus |
| IMAU32078 | Xinjiang, China | Fermented cow milk | 2012 | KF148766 | 4 | 4 | 3 | 1 | 3 | 13 | 10 | | 9 | | ST40 | | CC3 | | L5 | | bulgaricus |
| IMAU32435 | Xinjiang, China | Fermented cow milk | 2012 | KF149110 | 4 | 4 | 3 | 1 | 3 | 13 | 10 | | 9 | | ST40 | | CC3 | | L5 | | bulgaricus |
| IMAU32141 | Xinjiang, China | Fermented cow milk | 2012 | KF148826 | 4 | 4 | 3 | 1 | 3 | 13 | 10 | | 11 | | ST46 | | CC3 | | L5 | | bulgaricus |
| IMAU32166 | Xinjiang, China | Fermented cow milk | 2012 | KF148850 | 4 | 4 | 3 | 1 | 3 | 15 | 10 | | 11 | | ST49 | | CC3 | | L5 | | bulgaricus |
| IMAU32298 | Xinjiang, China | Fermented cow milk | 2012 | KF148976 | 4 | 4 | 3 | 1 | 3 | 14 | 1 | | 11 | | ST58 | | CC3 | | L6 | | bulgaricus |
| IMAU32612 | Xinjiang, China | Fermented cow milk | 2012 | KF149283 | 4 | 4 | 3 | 1 | 3 | 15 | 1 | | 11 | | ST67 | | CC3 | | L6 | | bulgaricus |
| IMAU32003 | Xinjiang, China | Fermented cow milk | 2012 | KF148693 | 4 | 2 | 10 | 1 | 3 | 13 | 10 | | 9 | | ST36 | | CC4 | | L5 | | bulgaricus |
| IMAU32093 | Xinjiang, China | Fermented cow milk | 2012 | KF148779 | 4 | 2 | 10 | 1 | 3 | 13 | 10 | | 9 | | ST36 | | CC4 | | L5 | | bulgaricus |
| IMAU32187 | Xinjiang, China | Fermented cow milk | 2012 | KF148871 | 4 | 2 | 10 | 1 | 3 | 13 | 10 | | 9 | | ST36 | | CC4 | | L5 | | bulgaricus |
| IMAU32330 | Xinjiang, China | Fermented cow milk | 2012 | KF149008 | 4 | 2 | 10 | 1 | 3 | 13 | 10 | | 9 | | ST36 | | CC4 | | L5 | | bulgaricus |
| IMAU32071 | Xinjiang, China | Fermented cow milk | 2012 | KF148759 | 4 | 2 | 10 | 1 | 10 | 13 | 10 | | 9 | | ST38 | | CC4 | | L5 | | bulgaricus |
| IMAU32072 | Xinjiang, China | Fermented cow milk | 2012 | KF148760 | 4 | 2 | 10 | 1 | 10 | 13 | 10 | | 9 | | ST38 | | CC4 | | L5 | | bulgaricus |
| IMAU32099 | Xinjiang, China | Fermented cow milk | 2012 | KF148785 | 4 | 2 | 10 | 1 | 10 | 13 | 10 | | 9 | | ST38 | | CC4 | | L5 | | bulgaricus |
| IMAU32121 | Xinjiang, China | Fermented cow milk | 2012 | KF148806 | 4 | 2 | 10 | 1 | 10 | 13 | 10 | | 9 | | ST38 | | CC4 | | L5 | | bulgaricus |
| IMAU32127 | Xinjiang, China | Fermented cow milk | 2012 | KF148812 | 4 | 2 | 10 | 1 | 10 | 13 | 10 | | 9 | | ST38 | | CC4 | | L5 | | bulgaricus |
| IMAU32481 | Xinjiang, China | Fermented cow milk | 2012 | KF149156 | 4 | 2 | 10 | 1 | 10 | 13 | 10 | | 9 | | ST38 | | CC4 | | L5 | | bulgaricus |
| IMAU32503 | Xinjiang, China | Fermented cow milk | 2012 | KF149177 | 4 | 2 | 10 | 1 | 10 | 13 | 10 | | 9 | | ST38 | | CC4 | | L5 | | bulgaricus |
| IMAU32556 | Xinjiang, China | Fermented cow milk | 2012 | KF149228 | 4 | 2 | 10 | 1 | 10 | 13 | 10 | | 9 | | ST38 | | CC4 | | L5 | | bulgaricus |
| IMAU32315 | Xinjiang, China | Fermented cow milk | 2012 | KF148993 | 4 | 2 | 3 | 1 | 10 | 13 | 10 | | 9 | | ST59 | | CC4 | | L5 | | bulgaricus |
| IMAU32477 | Xinjiang, China | Fermented cow milk | 2012 | KF149152 | 4 | 2 | 3 | 1 | 10 | 13 | 10 | | 9 | | ST59 | | CC4 | | L5 | | bulgaricus |
| IMAU32143 | Xinjiang, China | Fermented cow milk | 2012 | KF148828 | 9 | 2 | 11 | 1 | 3 | 13 | 10 | | 10 | | ST47 | | CC5 | | L5 | | bulgaricus |
| IMAU32146 | Xinjiang, China | Fermented cow milk | 2012 | KF148831 | 9 | 2 | 11 | 1 | 3 | 13 | 10 | | 10 | | ST47 | | CC5 | | L5 | | bulgaricus |
| IMAU32412 | Xinjiang, China | Fermented cow milk | 2012 | KF149088 | 9 | 2 | 11 | 1 | 3 | 13 | 10 | | 10 | | ST47 | | CC5 | | L5 | | bulgaricus |
| IMAU32217 | Xinjiang, China | Fermented cow milk | 2012 | KF148901 | 9 | 2 | 12 | 1 | 3 | 15 | 10 | | 10 | | ST53 | | CC5 | | L5 | | bulgaricus |
| IMAU32219 | Xinjiang, China | Fermented cow milk | 2012 | KF148903 | 9 | 2 | 12 | 1 | 3 | 15 | 10 | | 10 | | ST53 | | CC5 | | L5 | | bulgaricus |
| IMAU32262 | Xinjiang, China | Fermented cow milk | 2012 | KF148946 | 9 | 2 | 11 | 1 | 3 | 15 | 10 | | 10 | | ST55 | | CC5 | | L5 | | bulgaricus |
| IMAU32502 | Xinjiang, China | Fermented cow milk | 2012 | KF149176 | 9 | 2 | 11 | 1 | 3 | 15 | 10 | | 10 | | ST55 | | CC5 | | L5 | | bulgaricus |
| NBIMCC1273 | Bulgaria | Starter culture | - | - | 1 | 1 | 1 | 1 | 18 | 1 | 1 | | 1 | | ST110 | | doubleton | | L4 | | bulgaricus |
| NBIMCC1381 | Danmark | Starter culture | - | - | 2 | 2 | 3 | 1 | 1 | 3 | 1 | | 1 | | ST3 | | doubleton | | L4 | | bulgaricus |
| IMAU20525 | Mongolia | Fermented cow milk | 2009 | HM058244 | 4 | 4 | 6 | 1 | 9 | 6 | 1 | | 8 | | ST29 | | doubleton | | L1 | | bulgaricus |
| IMAU20527 | Mongolia | Fermented cow milk | 2009 | HM058246 | 4 | 4 | 6 | 1 | 9 | 6 | 5 | | 8 | | ST30 | | doubleton | | L1 | | bulgaricus |
| IMAU20528 | Mongolia | Fermented cow milk | 2009 | HM058247 | 4 | 4 | 6 | 1 | 9 | 6 | 5 | | 8 | | ST30 | | doubleton | | L1 | | bulgaricus |
| IMAU20639 | Mongolia | Fermented cow milk | 2009 | HM058353 | 4 | 8 | 9 | 1 | 9 | 6 | 4 | | 3 | | ST32 | | doubleton | | L1 | | bulgaricus |
| IMAU20641 | Mongolia | Fermented cow milk | 2009 | HM058355 | 4 | 4 | 9 | 1 | 9 | 6 | 4 | | 3 | | ST33 | | doubleton | | L1 | | bulgaricus |
| IMAU80385 | QSG of China^b^ | Fermented yak milk | 2009 | HM058654 | 3 | 3 | 3 | 17 | 2 | 4 | 4 | | 3 | | ST102 | | doubleton | | L2 | | bulgaricus |
| IMAU80266 | QSG of China^b^ | Fermented yak milk | 2009 | HM058549 | 3 | 3 | 3 | 4 | 2 | 4 | 4 | | 3 | | ST6 | | doubleton | | L2 | | bulgaricus |
| IMAU80698 | QSG of China^c^ | Fermented yak milk | 2009 | HM058878 | 15 | 4 | 5 | 10 | 3 | 11 | 12 | | 3 | | ST104 | | doubleton | | L1 | | bulgaricus |
| IMAU80699 | QSG of China^c^ | Fermented yak milk | 2009 | HM058879 | 15 | 4 | 5 | 10 | 2 | 11 | 12 | | 3 | | ST105 | | doubleton | | L1 | | bulgaricus |
| IMAU32065 | Xinjiang, China | Fermented cow milk | 2012 | KF148753 | 4 | 4 | 3 | 1 | 3 | 14 | 4 | | 6 | | ST37 | | doubleton | | L6 | | bulgaricus |
| IMAU32178 | Xinjiang, China | Fermented cow milk | 2012 | KF148862 | 4 | 4 | 3 | 1 | 10 | 14 | 4 | | 6 | | ST50 | | doubleton | | L6 | | bulgaricus |
| NBIMCC285 | Bulgaria | Home made yoghurt | - | - | 1 | 1 | 23 | 1 | 1 | 1 | 3 | | 1 | | ST111 | | singleton | | L4 | | bulgaricus |
| IMAU20310 | Mongolia | Fermented cow milk | 2009 | HM058040 | 1 | 2 | 2 | 1 | 8 | 9 | 3 | | 1 | | ST17 | | singleton | | L4 | | bulgaricus |
| IMAU20311 | Mongolia | Fermented cow milk | 2009 | HM058041 | 1 | 2 | 2 | 1 | 8 | 9 | 3 | | 1 | | ST17 | | singleton | | L4 | | bulgaricus |
| IMAU20312 | Mongolia | Fermented cow milk | 2009 | HM058042 | 1 | 2 | 2 | 1 | 8 | 9 | 3 | | 1 | | ST17 | | singleton | | L4 | | bulgaricus |
| IMAU20314 | Mongolia | Fermented cow milk | 2009 | HM058044 | 1 | 2 | 2 | 1 | 8 | 9 | 3 | | 1 | | ST17 | | singleton | | L4 | | bulgaricus |
| IMAU20435 | Mongolia | Fermented cow milk | 2009 | HM058159 | 4 | 4 | 3 | 1 | 9 | 6 | 4 | | 8 | | ST25 | | singleton | | L1 | | bulgaricus |
| IMAU20515 | Mongolia | Fermented cow milk | 2009 | HM058234 | 4 | 4 | 8 | 1 | 9 | 6 | 9 | | 3 | | ST28 | | singleton | | L1 | | bulgaricus |
| IMAU20516 | Mongolia | Fermented cow milk | 2009 | HM058235 | 4 | 4 | 8 | 1 | 9 | 6 | 9 | | 3 | | ST28 | | singleton | | L1 | | bulgaricus |
| IMAU20743 | Mongolia | Fermented cow milk | 2009 | HM058454 | 1 | 2 | 3 | 1 | 1 | 12 | 3 | | 1 | | ST34 | | singleton | | L4 | | bulgaricus |
| IMAU20176 | Mongolia | Fermented cow milk | 2009 | HM057913 | 5 | 4 | 5 | 1 | 3 | 6 | 5 | | 3 | | ST8 | | singleton | | L1 | | bulgaricus |
| IMAU20179 | Mongolia | Fermented cow milk | 2009 | HM057916 | 4 | 5 | 6 | 1 | 4 | 6 | 6 | | 3 | | ST9 | | singleton | | L1 | | bulgaricus |
| IMAU20489 | Mongolia | Fermented yak milk | 2009 | HM058208 | 4 | 4 | 3 | 1 | 9 | 11 | 5 | | 3 | | ST26 | | singleton | | L1 | | bulgaricus |
| IMAU40106 | QSG of China^a^ | Fermented yak milk | 2005 | FJ749379 | 3 | 4 | 15 | 10 | 3 | 4 | 12 | | 9 | | ST70 | | singleton | | L2 | | bulgaricus |
| IMAU40111 | QSG of China^a^ | Fermented yak milk | 2005 | FJ749383 | 4 | 3 | 5 | 11 | 2 | 11 | 4 | | 3 | | ST71 | | singleton | | L1 | | bulgaricus |
| IMAU80709 | QSG of China^c^ | Fermented yak milk | 2009 | HM058889 | 15 | 4 | 5 | 18 | 3 | 4 | 12 | | 9 | | ST106 | | singleton | | L2 | | bulgaricus |
| IMAU80798 | QSG of China^c^ | Fermented yak milk | 2009 | HM058963 | 1 | 15 | 2 | 1 | 1 | 1 | 3 | | 1 | | ST107 | | singleton | | L4 | | bulgaricus |
| IMAU80827 | QSG of China^c^ | Fermented yak milk | 2009 | HM058989 | 1 | 15 | 2 | 1 | 1 | 1 | 3 | | 1 | | ST107 | | singleton | | L4 | | bulgaricus |
| IMAU80828 | QSG of China^c^ | Fermented yak milk | 2009 | HM058990 | 3 | 4 | 3 | 10 | 3 | 4 | 4 | | 3 | | ST108 | | singleton | | L2 | | bulgaricus |
| IMAU80830 | QSG of China^c^ | Fermented yak milk | 2009 | HM058992 | 3 | 4 | 3 | 10 | 17 | 4 | 20 | | 3 | | ST109 | | singleton | | L2 | | bulgaricus |
| IMAU62217 | Tibet, China | Fermented yak milk | 2012 | KF149855 | 4 | 4 | 3 | 1 | 3 | 11 | 4 | | 14 | | ST100 | | singleton | | L1 | | bulgaricus |
| IMAU62081 | Tibet, China | Fermented yak milk | 2012 | KF149723 | 12 | 3 | 16 | 1 | 3 | 18 | 13 | | 4 | | ST73 | | singleton | | L3 | | bulgaricus |
| IMAU62090 | Tibet, China | Fermented yak milk | 2012 | KF149731 | 12 | 3 | 16 | 12 | 12 | 11 | 4 | | 4 | | ST74 | | singleton | | L3 | | bulgaricus |
| IMAU62091 | Tibet, China | Fermented yak milk | 2012 | KF149732 | 13 | 3 | 16 | 13 | 15 | 19 | 13 | | 4 | | ST75 | | singleton | | L3 | | bulgaricus |
| IMAU62093 | Tibet, China | Fermented yak milk | 2012 | KF149734 | 13 | 3 | 16 | 1 | 3 | 11 | 13 | | 4 | | ST76 | | singleton | | L3 | | bulgaricus |
| IMAU62103 | Tibet, China | Fermented yak milk | 2012 | KF149744 | 12 | 3 | 16 | 10 | 12 | 19 | 4 | | 4 | | ST77 | | singleton | | L3 | | bulgaricus |
| IMAU62105 | Tibet, China | Fermented yak milk | 2012 | KF149746 | 12 | 11 | 16 | 14 | 3 | 19 | 4 | | 4 | | ST78 | | singleton | | L3 | | bulgaricus |
| IMAU62110 | Tibet, China | Fermented yak milk | 2012 | KF149750 | 12 | 11 | 16 | 13 | 3 | 15 | 13 | | 4 | | ST79 | | singleton | | L3 | | bulgaricus |
| IMAU62111 | Tibet, China | Fermented yak milk | 2012 | KF149751 | 12 | 3 | 3 | 14 | 3 | 19 | 14 | | 4 | | ST80 | | singleton | | L3 | | bulgaricus |
| IMAU62115 | Tibet, China | Fermented yak milk | 2012 | KF149755 | 14 | 11 | 17 | 1 | 3 | 11 | 4 | | 4 | | ST81 | | singleton | | L1 | | bulgaricus |
| IMAU62121 | Tibet, China | Fermented yak milk | 2012 | KF149761 | 14 | 11 | 17 | 1 | 3 | 11 | 4 | | 4 | | ST81 | | singleton | | L1 | | bulgaricus |
| IMAU62125 | Tibet, China | Fermented yak milk | 2012 | KF149765 | 4 | 3 | 18 | 11 | 3 | 11 | 15 | | 13 | | ST82 | | singleton | | L1 | | bulgaricus |
| IMAU62139 | Tibet, China | Fermented yak milk | 2012 | KF149779 | 15 | 12 | 3 | 10 | 3 | 11 | 14 | | 14 | | ST83 | | singleton | | L1 | | bulgaricus |
| IMAU62141 | Tibet, China | Fermented yak milk | 2012 | KF149781 | 4 | 4 | 3 | 10 | 3 | 11 | 4 | | 4 | | ST84 | | singleton | | L1 | | bulgaricus |
| IMAU62142 | Tibet, China | Fermented yak milk | 2012 | KF149782 | 13 | 11 | 16 | 1 | 12 | 19 | 16 | | 4 | | ST85 | | singleton | | L3 | | bulgaricus |
| IMAU62150 | Tibet, China | Fermented yak milk | 2012 | KF149790 | 4 | 13 | 19 | 10 | 9 | 11 | 4 | | 14 | | ST86 | | singleton | | L1 | | bulgaricus |
| IMAU62169 | Tibet, China | Fermented yak milk | 2012 | KF149808 | 4 | 13 | 19 | 10 | 9 | 11 | 4 | | 14 | | ST86 | | singleton | | L1 | | bulgaricus |
| IMAU62152 | Tibet, China | Fermented yak milk | 2012 | KF149792 | 4 | 4 | 20 | 10 | 9 | 11 | 4 | | 14 | | ST87 | | singleton | | L1 | | bulgaricus |
| IMAU62153 | Tibet, China | Fermented yak milk | 2012 | KF149793 | 12 | 11 | 16 | 1 | 3 | 15 | 13 | | 9 | | ST88 | | singleton | | L3 | | bulgaricus |
| IMAU62159 | Tibet, China | Fermented yak milk | 2012 | KF149798 | 15 | 3 | 3 | 10 | 3 | 11 | 13 | | 4 | | ST89 | | singleton | | L1 | | bulgaricus |
| IMAU62160 | Tibet, China | Fermented yak milk | 2012 | KF149799 | 16 | 14 | 16 | 13 | 3 | 11 | 17 | | 15 | | ST90 | | singleton | | L3 | | bulgaricus |
| IMAU62161 | Tibet, China | Fermented yak milk | 2012 | KF149800 | 4 | 4 | 3 | 15 | 9 | 20 | 5 | | 3 | | ST91 | | singleton | | L1 | | bulgaricus |
| IMAU62164 | Tibet, China | Fermented yak milk | 2012 | KF149803 | 4 | 11 | 3 | 10 | 3 | 11 | 4 | | 9 | | ST92 | | singleton | | L1 | | bulgaricus |
| IMAU62165 | Tibet, China | Fermented yak milk | 2012 | KF149804 | 15 | 4 | 17 | 10 | 3 | 11 | 18 | | 9 | | ST93 | | singleton | | L1 | | bulgaricus |
| IMAU62168 | Tibet, China | Fermented yak milk | 2012 | KF149807 | 4 | 3 | 5 | 1 | 3 | 11 | 14 | | 14 | | ST94 | | singleton | | L1 | | bulgaricus |
| IMAU62185 | Tibet, China | Fermented yak milk | 2012 | KF149824 | 13 | 14 | 3 | 10 | 15 | 21 | 4 | | 4 | | ST95 | | singleton | | L3 | | bulgaricus |
| IMAU62186 | Tibet, China | Fermented yak milk | 2012 | KF149825 | 13 | 3 | 21 | 10 | 15 | 11 | 4 | | 4 | | ST96 | | singleton | | L3 | | bulgaricus |
| IMAU62187 | Tibet, China | Fermented yak milk | 2012 | KF149826 | 13 | 3 | 21 | 10 | 15 | 11 | 4 | | 4 | | ST96 | | singleton | | L3 | | bulgaricus |
| IMAU62193 | Tibet, China | Fermented yak milk | 2012 | KF149831 | 15 | 4 | 5 | 10 | 12 | 22 | 12 | | 3 | | ST97 | | singleton | | L1 | | bulgaricus |
| IMAU62203 | Tibet, China | Fermented yak milk | 2012 | KF149841 | 12 | 3 | 16 | 10 | 12 | 11 | 16 | | 9 | | ST98 | | singleton | | L3 | | bulgaricus |
| IMAU62204 | Tibet, China | Fermented yak milk | 2012 | KF149842 | 15 | 4 | 3 | 10 | 3 | 11 | 14 | | 9 | | ST99 | | singleton | | L1 | | bulgaricus |
| IMAU32096 | Xinjiang, China | Fermented cow milk | 2012 | KF148782 | 4 | 8 | 12 | 1 | 3 | 15 | 10 | | 10 | | ST42 | | singleton | | L5 | | bulgaricus |
| IMAU32104 | Xinjiang, China | Fermented cow milk | 2012 | KF148790 | 4 | 4 | 3 | 1 | 3 | 16 | 4 | | 11 | | ST43 | | singleton | | L6 | | bulgaricus |
| IMAU32270 | Xinjiang, China | Fermented cow milk | 2012 | KF148952 | 4 | 4 | 3 | 1 | 3 | 16 | 4 | | 11 | | ST43 | | singleton | | L6 | | bulgaricus |
| IMAU32115 | Xinjiang, China | Fermented cow milk | 2012 | KF148800 | 4 | 4 | 10 | 1 | 12 | 13 | 10 | | 9 | | ST45 | | singleton | | L5 | | bulgaricus |
| IMAU32164 | Xinjiang, China | Fermented cow milk | 2012 | KF148848 | 10 | 4 | 3 | 1 | 3 | 15 | 4 | | 11 | | ST48 | | singleton | | L6 | | bulgaricus |
| IMAU32276 | Xinjiang, China | Fermented cow milk | 2012 | KF148957 | 4 | 4 | 10 | 1 | 3 | 14 | 10 | | 9 | | ST56 | | singleton | | L6 | | bulgaricus |
| IMAU32278 | Xinjiang, China | Fermented cow milk | 2012 | KF148959 | 4 | 4 | 10 | 1 | 3 | 14 | 10 | | 9 | | ST56 | | singleton | | L6 | | bulgaricus |
| IMAU32279 | Xinjiang, China | Fermented cow milk | 2012 | KF148960 | 10 | 4 | 3 | 1 | 3 | 16 | 1 | | 6 | | ST57 | | singleton | | L6 | | bulgaricus |
| IMAU32355 | Xinjiang, China | Fermented cow milk | 2012 | KF149033 | 9 | 4 | 3 | 1 | 3 | 4 | 4 | | 6 | | ST62 | | singleton | | L6 | | bulgaricus |
| IMAU32357 | Xinjiang, China | Fermented cow milk | 2012 | KF149035 | 9 | 4 | 3 | 1 | 3 | 4 | 4 | | 6 | | ST62 | | singleton | | L6 | | bulgaricus |
| IMAU32358 | Xinjiang, China | Fermented cow milk | 2012 | KF149036 | 9 | 4 | 3 | 1 | 3 | 4 | 4 | | 6 | | ST62 | | singleton | | L6 | | bulgaricus |
| IMAU32359 | Xinjiang, China | Fermented cow milk | 2012 | KF149037 | 9 | 4 | 3 | 1 | 3 | 4 | 4 | | 6 | | ST62 | | singleton | | L6 | | bulgaricus |
| IMAU32368 | Xinjiang, China | Fermented cow milk | 2012 | KF149046 | 9 | 2 | 3 | 1 | 3 | 15 | 10 | | 9 | | ST64 | | singleton | | L5 | | bulgaricus |
| IMAU32370 | Xinjiang, China | Fermented cow milk | 2012 | KF149048 | 4 | 2 | 3 | 1 | 3 | 15 | 1 | | 3 | | ST65 | | singleton | | L6 | | bulgaricus |
| IMAU32371 | Xinjiang, China | Fermented cow milk | 2012 | KF149049 | 4 | 2 | 3 | 1 | 3 | 15 | 1 | | 3 | | ST65 | | singleton | | L6 | | bulgaricus |
| IMAU80318 | QSG of China^b^ | Qula | 2009 | HM058598 | 11 | 10 | 22 | 16 | 14 | 17 | 11 | | 12 | | ST101 | | singleton | | - | | other ssp |
| IMAU80319 | QSG of China^b^ | Qula | 2009 | HM058599 | 11 | 10 | 22 | 16 | 14 | 17 | 11 | | 12 | | ST101 | | singleton | | - | | other ssp |
| IMAU80314 | QSG of China^b^ | Whey | 2009 | HM058594 | 11 | 10 | 22 | 16 | 14 | 17 | 11 | | 12 | | ST101 | | singleton | | - | | other ssp |
| IMAU80423 | QSG of China^b^ | Qula | 2009 | HM217994 | 11 | 10 | 22 | 16 | 16 | 17 | 19 | | 16 | | ST103 | | singleton | | - | | other ssp |
| IMAU40156 | QSG of China^a^ | Fermented goat milk | 2005 | FJ915694 | 6 | 6 | 7 | 5 | 5 | 7 | 7 | | 1 | | ST11 | | doubleton | | - | | other ssp |
| IMAU40157 | QSG of China^a^ | Fermented goat milk | 2005 | FJ915695 | 6 | 6 | 7 | 5 | 5 | 7 | 7 | | 1 | | ST11 | | doubleton | | - | | other ssp |
| IMAU40166 | QSG of China^a^ | Fermented goat milk | 2005 | FJ915703 | 6 | 6 | 7 | 5 | 5 | 7 | 7 | | 1 | | ST11 | | doubleton | | - | | other ssp |
| IMAU40167 | QSG of China^a^ | Fermented goat milk | 2005 | FJ915704 | 6 | 6 | 7 | 5 | 5 | 7 | 7 | | 1 | | ST11 | | doubleton | | - | | other ssp |
| IMAU40065 | QSG of China^a^ | Fermented yak milk | 2005 | FJ749340 | 6 | 6 | 7 | 5 | 5 | 7 | 7 | | 1 | | ST11 | | doubleton | | - | | other ssp |
| IMAU40073 | QSG of China^a^ | Fermented yak milk | 2005 | FJ749348 | 6 | 6 | 7 | 5 | 5 | 7 | 7 | | 1 | | ST11 | | doubleton | | - | | other ssp |
| IMAU40077 | QSG of China^a^ | Fermented yak milk | 2005 | FJ749352 | 6 | 6 | 7 | 5 | 5 | 7 | 7 | | 1 | | ST11 | | doubleton | | - | | other ssp |
| IMAU40078 | QSG of China^a^ | Fermented yak milk | 2005 | FJ749353 | 6 | 6 | 7 | 5 | 5 | 7 | 7 | | 1 | | ST11 | | doubleton | | - | | other ssp |
| IMAU32212 | Xinjiang, China | Fermented cow milk | 2012 | KF148896 | 6 | 6 | 7 | 5 | 5 | 7 | 7 | | 1 | | ST11 | | doubleton | | - | | other ssp |
| IMAU90013 | Russia | Fermented cow milk | 2012 | KC836531 | 4 | 10 | 26 | 1 | 14 | 5 | 4 | | 4 | | ST117 | | singleton | | - | | other ssp |
| IMAU94251 | Russia | Fermented cow milk | 2012 | KF149626 | 11 | 10 | 26 | 1 | 21 | 5 | 4 | | 4 | | ST118 | | singleton | | - | | other ssp |
| IMAU20287 | Mongolia | Fermented cow milk | 2009 | HM058018 | 7 | 7 | 2 | 6 | 6 | 8 | 8 | | 7 | | ST15 | | singleton | | - | | other ssp |
| IMAU90053 | Russia | Fermented cow milk | 2012 | KC836571 | 11 | 10 | 14 | 9 | 14 | 17 | 11 | | 12 | | ST69 | | singleton | | - | | other ssp |
| IMAU90230 | Russia | Fermented cow milk | 2012 | KC836740 | 11 | 10 | 14 | 9 | 14 | 17 | 11 | | 12 | | ST69 | | singleton | | - | | other ssp |
| IMAU90231 | Russia | Fermented cow milk | 2012 | KC836741 | 11 | 10 | 14 | 9 | 14 | 17 | 11 | | 12 | | ST69 | | singleton | | - | | other ssp |
| IMAU92008 | Russia | Fermented cow milk | 2012 | KF149402 | 11 | 10 | 14 | 9 | 14 | 17 | 11 | | 12 | | ST69 | | singleton | | - | | other ssp |
| IMAU92009 | Russia | Fermented cow milk | 2012 | KF149403 | 11 | 10 | 14 | 9 | 14 | 17 | 11 | | 12 | | ST69 | | singleton | | - | | other ssp |
| IMAU92011 | Russia | Fermented cow milk | 2012 | KF149405 | 11 | 10 | 14 | 9 | 14 | 17 | 11 | | 12 | | ST69 | | singleton | | - | | other ssp |
| IMAU92017 | Russia | Fermented cow milk | 2012 | KF149411 | 11 | 10 | 14 | 9 | 14 | 17 | 11 | | 12 | | ST69 | | singleton | | - | | other ssp |
| IMAU92018 | Russia | Fermented cow milk | 2012 | KF149412 | 11 | 10 | 14 | 9 | 14 | 17 | 11 | | 12 | | ST69 | | singleton | | - | | other ssp |
| IMAU92019 | Russia | Fermented cow milk | 2012 | KF149413 | 11 | 10 | 14 | 9 | 14 | 17 | 11 | | 12 | | ST69 | | singleton | | - | | other ssp |
| IMAU92020 | Russia | Fermented cow milk | 2012 | KF149414 | 11 | 10 | 14 | 9 | 14 | 17 | 11 | | 12 | | ST69 | | singleton | | - | | other ssp |
| IMAU92021 | Russia | Fermented cow milk | 2012 | KF149415 | 11 | 10 | 14 | 9 | 14 | 17 | 11 | | 12 | | ST69 | | singleton | | - | | other ssp |
| IMAU92022 | Russia | Fermented cow milk | 2012 | KF149416 | 11 | 10 | 14 | 9 | 14 | 17 | 11 | | 12 | | ST69 | | singleton | | - | | other ssp |
| IMAU92024 | Russia | Fermented cow milk | 2012 | KF149418 | 11 | 10 | 14 | 9 | 14 | 17 | 11 | | 12 | | ST69 | | singleton | | - | | other ssp |
| IMAU92026 | Russia | Fermented cow milk | 2012 | KF149420 | 11 | 10 | 14 | 9 | 14 | 17 | 11 | | 12 | | ST69 | | singleton | | - | | other ssp |
| IMAU92027 | Russia | Fermented cow milk | 2012 | KF149421 | 11 | 10 | 14 | 9 | 14 | 17 | 11 | | 12 | | ST69 | | singleton | | - | | other ssp |
| IMAU92028 | Russia | Fermented cow milk | 2012 | KF149422 | 11 | 10 | 14 | 9 | 14 | 17 | 11 | | 12 | | ST69 | | singleton | | - | | other ssp |
| IMAU92029 | Russia | Fermented cow milk | 2012 | KF149423 | 11 | 10 | 14 | 9 | 14 | 17 | 11 | | 12 | | ST69 | | singleton | | - | | other ssp |
| IMAU92030 | Russia | Fermented cow milk | 2012 | KF149424 | 11 | 10 | 14 | 9 | 14 | 17 | 11 | | 12 | | ST69 | | singleton | | - | | other ssp |
| IMAU92031 | Russia | Fermented cow milk | 2012 | KF149425 | 11 | 10 | 14 | 9 | 14 | 17 | 11 | | 12 | | ST69 | | singleton | | - | | other ssp |
| IMAU92033 | Russia | Fermented cow milk | 2012 | KF149427 | 11 | 10 | 14 | 9 | 14 | 17 | 11 | | 12 | | ST69 | | singleton | | - | | other ssp |
| IMAU92066 | Russia | Fermented cow milk | 2012 | KF149459 | 11 | 10 | 14 | 9 | 14 | 17 | 11 | | 12 | | ST69 | | singleton | | - | | other ssp |
| IMAU92068 | Russia | Fermented cow milk | 2012 | KF149461 | 11 | 10 | 14 | 9 | 14 | 17 | 11 | | 12 | | ST69 | | singleton | | - | | other ssp |
| IMAU94262 | Russia | Fermented cow milk | 2012 | KF149636 | 11 | 10 | 14 | 9 | 14 | 17 | 11 | | 12 | | ST69 | | singleton | | - | | other ssp |
| IMAU40080 | QSG of China^a^ | Fermented yak milk | 2005 | FJ749355 | 11 | 10 | 14 | 9 | 14 | 17 | 11 | | 12 | | ST69 | | singleton | | - | | other ssp |
| IMAU40168 | QSG of China^a^ | Fermented goat milk | 2005 | FJ915705 | 6 | 6 | 7 | 5 | 3 | 7 | 7 | | 1 | | ST72 | | doubleton | | - | | other ssp |
| 2038^*^ | Japan | Starter culture | - | - | 1 | 1 | 1 | 1 | 1 | 1 | 1 | | 1 | | ST1 | | doubleton | | L4 | | bulgaricus |
| ATCC11842^T,*^ | Bulgaria | bulgarian yogurt | 1919 | - | 1 | 2 | 2 | 2 | 1 | 2 | 2 | | 2 | | ST2 | | singleton | | L4 | | bulgaricus |
| ATCC BAA-365^*^ | France | Starter culture | - | - | 2 | 2 | 3 | 1 | 1 | 3 | 1 | | 1 | | ST3 | | doubleton | | L4 | | bulgaricus |
| CNCM1519^*^ | America | Starter culture | - | - | 1 | 2 | 2 | 3 | 1 | 1 | 3 | | 1 | | ST4 | | singleton | | L4 | | bulgaricus |
| CNCM1632^*^ | America | Starter culture | - | - | 2 | 2 | 4 | 1 | 1 | 3 | 1 | | 1 | | ST5 | | doubleton | | L4 | | bulgaricus |
| ND02^*^ | China | Fermented yak milk | 2005 | - | 6 | 6 | 7 | 5 | 5 | 7 | 7 | | 1 | | ST11 | | doubleton | | - | | other ssp |
| ZN7a-9^T,*^ | BurkinaFaso | Indigenous African fermented beverage (Dolo) | 2013 | - | 19 | 18 | 25 | 21 | 20 | 25 | 24 | | 19 | | ST115 | | singleton | | - | | other ssp |
| DSM15996^T,*^ | India | traditional dairy fermented product (Dahi type) | 2005 | - | 17 | 16 | 24 | 19 | 19 | 23 | 21 | | 17 | | ST112 | | singleton | | - | | other ssp |
| DSM20072^T,*^ | - | emmental cheese | 1919 | - | 18 | 17 | 14 | 20 | 5 | 24 | 22 | | 18 | | ST113 | | singleton | | - | | other ssp |
| JCM1012^T,*^ | - | sour grain mash | 1896 | - | 20 | 19 | 27 | 5 | 14 | 26 | 23 | | 20 | | ST114 | | singleton | | - | | other ssp |
| DSM24966^T^ | Japan | traditional Japanese pickle(sunki) | 2012 | - | 21 | 20 | 24 | 22 | 22 | 27 | 25 | | 21 | | ST119 | | singleton | | - | | other ssp |
| Note: ^a^: Qinghai, China: ^b^: Sichuan, China; ^c^: GanSu, China; | | |  |  |  |  |  |  |  |  |  | |  | |  | |  | |  | |  |
| ^T^: Type strains of *L. delbrueckii* (ATCC11842(NC_008054): ssp.*bulgaricus*; ZN7a-9(ALPY01000001): ssp.*jakobsenii*; DSM15996(AZFL00000000): ssp.*indicus*; DSM20072(AZDE00000000): ssp.*lactis*; JCM1012(NZ_BALP01000000): ssp.*delbrueckii*; DSM24966: ssp.*sunkii*) | | | | | | | | | | | | | | | | | | | | | |
| *: The fragment sequences of 8 loci on these strains were retrieved from NCBI (http://www.ncbi.nlm.nih.gov/genome/genomes/514). | | | | | | | | | | |  |  | |  | |  | |  | |  | |
